# CCDC25 suppresses clear cell renal cell carcinoma progression by LATS1/YAP-mediated regulation of the hippo pathway

**DOI:** 10.1186/s12935-024-03318-0

**Published:** 2024-04-03

**Authors:** Hongpei Tan, Jiahao Liu, Yanan Li, Ze Mi, Baiying Liu, Pengfei Rong

**Affiliations:** 1 Department of Radiology, Third Xiangya Hospital, Central South University, Changsha, 410000 China; 2 Department of Gastrointestinal Surgery, Third Xiangya Hospital, Central South University, Changsha, China

**Keywords:** CCDC25, Clear cell renal cell carcinoma, Hippo, Prognosis, YAP (Yes-associated protein)

## Abstract

**Background:**

Clear cell renal cell carcinoma (ccRCC) is one of the most prevalent renal cancers, and the molecular mechanisms underlying its progression are still not fully understood. The expression of CCDC25, a notably underexpressed gene in many tumors, has been understudied in ccRCC. This research aims to explore the role of CCDC25 in ccRCC's clinical outcomes and uncover the molecular pathways influenced by it.

**Methods:**

A multi-tiered approach was adopted involving bioinformatic analysis, tissue sample evaluation, in vitro and in vivo experiments. CCDC25 expression levels in tumor vs. normal tissues were quantified using Western blot and immunofluorescence studies. Cell proliferation and migration were analyzed using CCK8, EDU, Transwell assays, and wound healing assays. RNA sequencing was performed to elucidate the molecular pathways affected, followed by detailed protein–protein interaction studies and mouse xenograft models.

**Results:**

CCDC25 was predominantly underexpressed in ccRCC tumors and associated with advanced clinical stages and poor prognosis. Overexpression of CCDC25 in renal cancer cell lines resulted in reduced proliferation and migration. RNA sequencing revealed significant alterations in the Hippo pathway. Overexpression of CCDC25 inhibited the expression of downstream Hippo pathway proteins ITGA3 and CCND1 and promoted YAP phosphorylation. Mechanistic studies showed that CCDC25 interacts with YAP and influences YAP phosphorylation through LATS1. In vivo, CCDC25 overexpression inhibited tumor growth and promoted apoptosis.

**Conclusion:**

CCDC25 acts as a potential tumor suppressor in ccRCC by inhibiting cell proliferation and migration, potentially through regulating the Hippo signaling pathway. These findings highlight the potential of CCDC25 as a therapeutic target in ccRCC treatment.

**Graphical Abstract:**

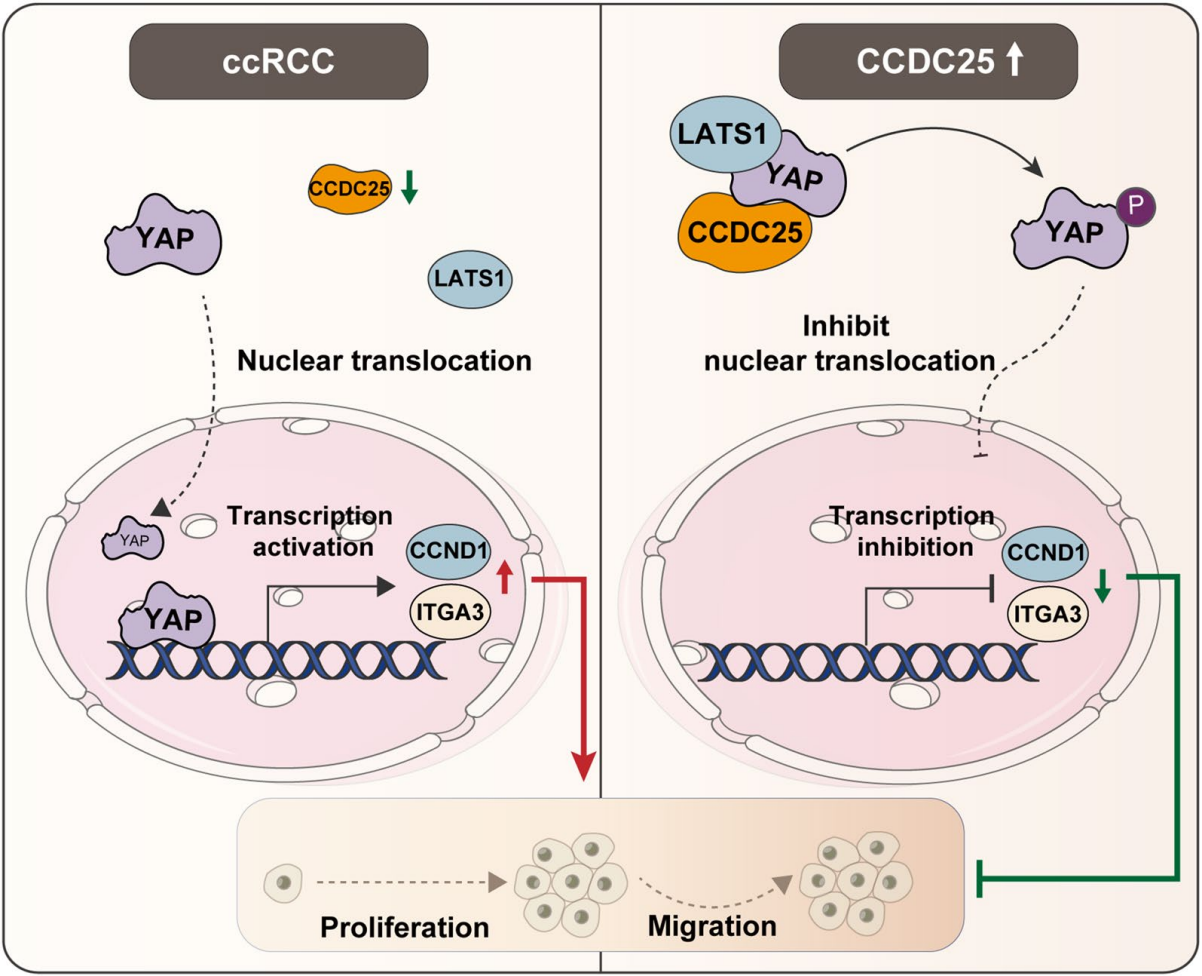

**Supplementary Information:**

The online version contains supplementary material available at 10.1186/s12935-024-03318-0.

## Introduction

Renal cell carcinoma (RCC) is a common clinical malignancy of the urinary system, accounting for approximately 90% of kidney cancers tumor [1]. Clear cell renal cell carcinoma (ccRCC) is the most common subtype of RCC, characterized by aggressive behavior and a high propensity for metastasis [2–4]. ccRCC may be easily misdiagnosed due to its insidious onset and lack of specific clinical symptoms in the early stage [5]. Despite significant advancements in diagnosis and treatment, including targeted therapies and immunotherapy, the prognosis for patients with advanced or metastatic ccRCC remains poor [6]. Therefore, understanding the molecular mechanisms driving ccRCC progression is paramount in developing new and more effective therapeutic strategies.

The coiled-coil domain-containing (CCDC) protein family encompasses a diverse group of proteins participating in various biological processes [7]. Recent research has implicated several CCDC proteins in cancer development and progression, suggesting that members of this family may serve as valuable therapeutic targets [8–10]. One such protein, CCDC25, has been identified to be associated with neutrophil extracellular trap binding [11], emerges as a novel candidate in cancer research, yet its oncogenic role in ccRCC remains underexplored. Given the pivotal processes influenced by CCDC proteins, CCDC25's involvement in ccRCC could offer new therapeutic insights.

In this study, we aimed to investigate the expression of CCDC25 in ccRCC and its potential association with clinical outcomes. We also sought to explore the functional significance of CCDC25 in ccRCC by evaluating its impact on cell proliferation, migration, and apoptosis. Additionally, through RNA sequencing and pathway analysis, we aimed to uncover the potential molecular mechanisms underlying the effects of CCDC25 on ccRCC. By elucidating the role of CCDC25 in ccRCC, we hope our study could not only deepen our understanding of ccRCC's molecular underpinnings but also propel the development of novel treatment paradigms.

## Methods

### Bioinformatics mining of CCDC25 expression and prognostic value

We obtained the expression level of CCDC25 in The Cancer Genome Atlas (TCGA) pan-cancer cohort using the TIMER2 database (http://timer.compgenomics.org/). We downloaded transcriptome data and clinical information of ccRCC from the TCGA (https://portal.gdc.cancer.gov/) database, including 542 ccRCC samples and 72 normal samples, processed using the Bioconductor package in the R statistical environment. GEPIA (http://gepia.cancer-pku.cn/index.html) was used to analyze the prognostic value of CCDC25 in ccRCC. The relationship between the expression of CCDC25 and the tumor grade of ccRCC was analyzed by UALCAN (https://ualcan.path.uab.edu/).

### Tissue samples

Human ccRCC tissues and adjacent normal tissues were acquired from 7 patients diagnosed as ccRCC in the Department of Urology, third Xiangya hospital. The ethical committee of third xiangya Hospital approved this study. All patients agreed that their samples could be utilized in the experimental studies and publication.

### Cell lines

Several renal cancer cell lines (caki-1, caki-2, 786–0, A498) and normal renal cell lines (HK2,293 T) were used for subsequent experiments [12, 13], obtained from the American Type Culture Collection (ATCC), USA. All cells were cultured at 37 °C and 5% CO_2_ in the appropriate medium, the details was listed in Additional file 1. We infected Caki-1 and 786–0 cells with CCDC25 overexpression lentivirus, The stable expression cell lines were screened by adding purinomycin at 2ug/mL respectively. The corresponding gene was knocked down using shRNA and Lipofectamine3000. The knockdown and overexpression efficiency were then verified by Western Blot.

### Cell proliferation

Cell proliferation was assessed using the CCK8 assay and EDU staining in ccRCC cell lines.

### Migration assays

The effect of CCDC25 overexpression on cell migration was evaluated by Transwell assay and scratch healing assay. 1 × 10^5^ cells were inoculated in a Transwell chamber with a pore size of 8.0 μm and suspended in 150 UL serum-free medium. The corresponding medium of 600ul with 20%FBS was added to the lower chamber, incubated at 37℃ for 24 h, then fixed, stained, and photographed. As for the scratch healing assay, the cells were seeded in a 6-well plate with the complete medium. When the cells grow to 75% alignment, the cells are damaged with the tip of a sterile pipette and the cell debris is removed with PBS washing. Next, serum-free medium was added to the six-well plate and cultured in an incubator for 48 h. Take pictures and assess the closing rate.

### RNA sequencing and pathway analysis

RNA transcriptome sequencing analysis was conducted on 786–0 cells overexpressing CCDC25 and normal 786–0 cells. Gene expression differential analysis was conducted utilizing the 'limma' R package, employing an adjusted p-value of < 0.05 as the selection threshold. For pathway enrichment analysis, the Kyoto Encyclopedia of Genes and Genomes (KEGG) analysis was performed using the 'clusterprofiler' R package, with the 'ggplot2' R package facilitating the mapping of results. A significance threshold for KEGG pathway enrichment was established at p < 0.05.

### Western Blot, Co-IP, Nuclear Extraction

The detailed protocols for Western blot, Co-immunoprecipitation, Nuclear Extraction are described in Additional file 1.

### Luciferase reporter assay

Cells were grown in 24-well culture plates and co-transfected with mixtures of the required reporter plasmids. 24 h after transfection, the cells were cleaved at room temperature for 10 min. The 20ul supernatant obtained after 5 min centrifugation at 12,000 rpm was used for determination.

### Mouse xenograft model

The 786–0 cells were washed with PBS to obtain a 1 × 10^7^ cell /ml suspension. Adult male nude mice were used to establish tumor models. The mice were anesthetized with isoflurane, and a cell suspension (100 μL) was injected subcutaneously into the right back of each mouse [14, 15]. Mouse tumor size was measured every 2 days using vernier calipers. Once the tumors reached an appropriate size, the mice were euthanized and the tumors were collected for further analysis, such as histological examination, immunohistochemistry, and immunofluorescence.

### Statistical analysis

The experimental results were all analyzed using SPSS 26.0 statistical software. All quantitative results are presented as mean ± standard deviation (SD). Differences among multiple groups were evaluated using a one-way analysis of variance (ANOVA). R programming language (version 4.1.0; R Foundation) was used to evaluate the data retrived from TCGA database. The levels of significance were denoted as: *p < 0.05, **p < 0.01, ***p < 0.001, and ****p < 0.0001.

## Results

### The expression of ccdc25 is decreased in the tumor and is associated with the clinical stage and prognosis of ccRCC patients

**Fig. 1:**
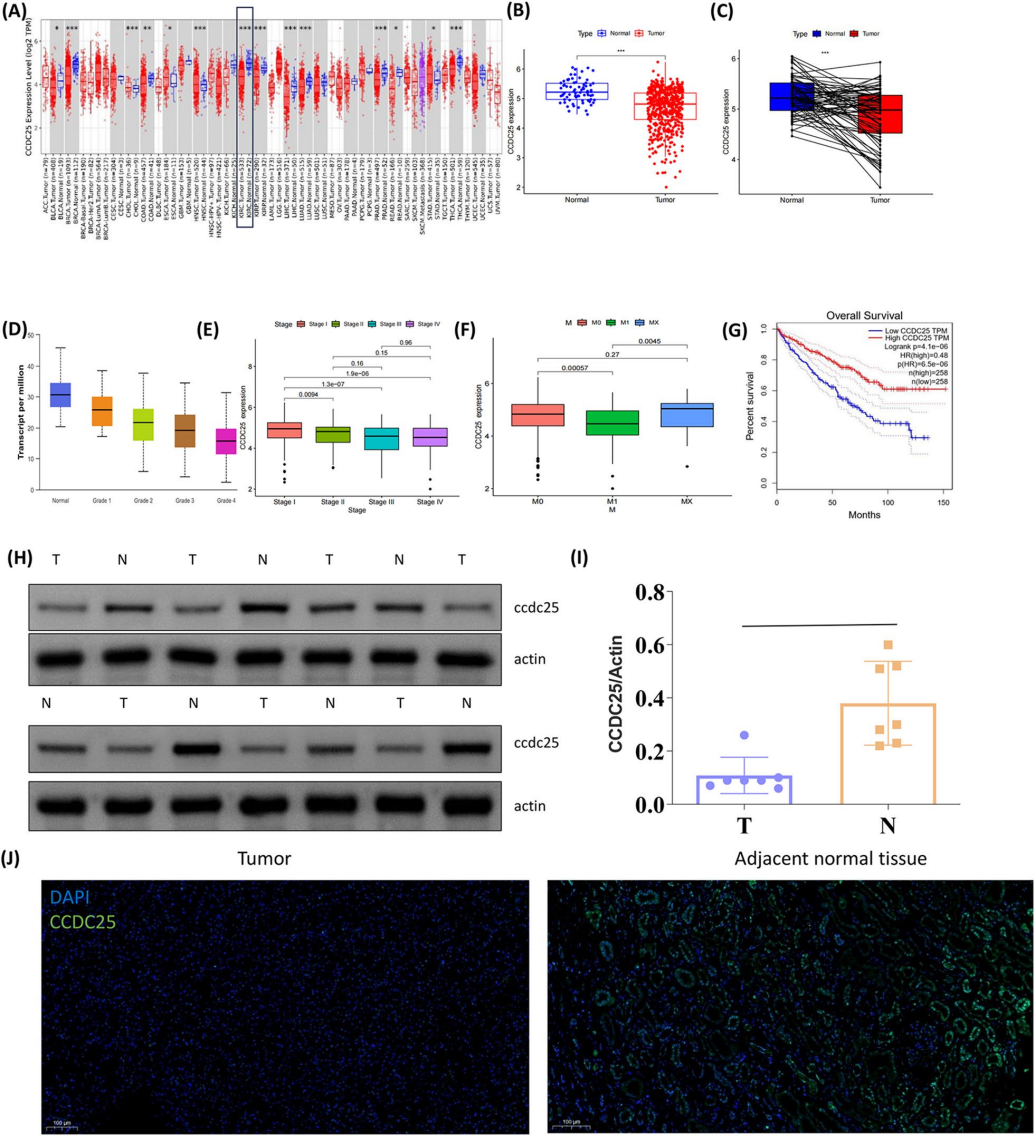
Expression of CCDC25 in ccRCC. **A** CCDC25 expression indifferent types of cancer was investigated with the TIMER database. **B** Analysis of CCDC25 expression in ccRCC and adjacent normal tissues in the TCGA database. **C** TCGA database and statistical analyses of CCDC25 expression in 72 pairs of ccRCC tissues and adjacent normal tissues. Box plots evaluating CCDC25 expression among different groups of tumor grade **D**, stage **E** and metastasis **F** of ccRCC patients based on clinical parameters of TCGA database. **G** Survival curves using the GEPIA database are shown for OS. **H** Western blot was performed in ccRCC and normal renal tissues to analyse the expression of CCDC25. **I** Quantitative analysis of CCDC25. **J** Immunofluorescence was used to detect CCDC25 expression in tumor tissues and adjacent normal renal tissues. Scar bar, 100 μm

We first analyzed the expression of CCDC25 across a spectrum of cancers, observing that CCDC25 is predominantly underexpressed in most tumors. In ccRCC (clear cell renal cell carcinoma), the expression of CCDC25 within tumors is markedly lower than in normal tissues (Fig. 1A, B). Further paired analysis revealed that the expression of CCDC25 in tumor regions is significantly lower within the same patient than in normal tissues (Fig. 1C). When categorizing patients based on tumor grade and staging, we found that as the tumor grade/stage increases, CCDC25 expression gradually diminishes (Fig. 1D, E). Notably, we discovered that patients with distant metastases exhibit substantially lower levels of CCDC25 expression compared to those without metastases (Fig. 1F). Additional survival analysis indicates that ccRCC patients with high CCDC25 expression have a significantly improved overall survival rate (Fig. 1G). These findings suggest that CCDC25 may play an important role in the disease progression in ccRCC patients.

We collected tumor tissues and corresponding adjacent non-tumor tissues from ccRCC (clear cell renal cell carcinoma) patients. Using Western blot (WB) analysis, we measured the expression levels of CCDC25 and found that its expression was notably lower in tumor tissues compared to the adjacent normal tissues (Fig. 1H, I). Further immunofluorescence studies also demonstrated that the expression of CCDC25 within tumor tissues was significantly lower than that in the adjacent normal renal tissues (Fig. 1J).

### CCDC25 has inhibitory effect on renal cancer cells

**Fig. 2:**
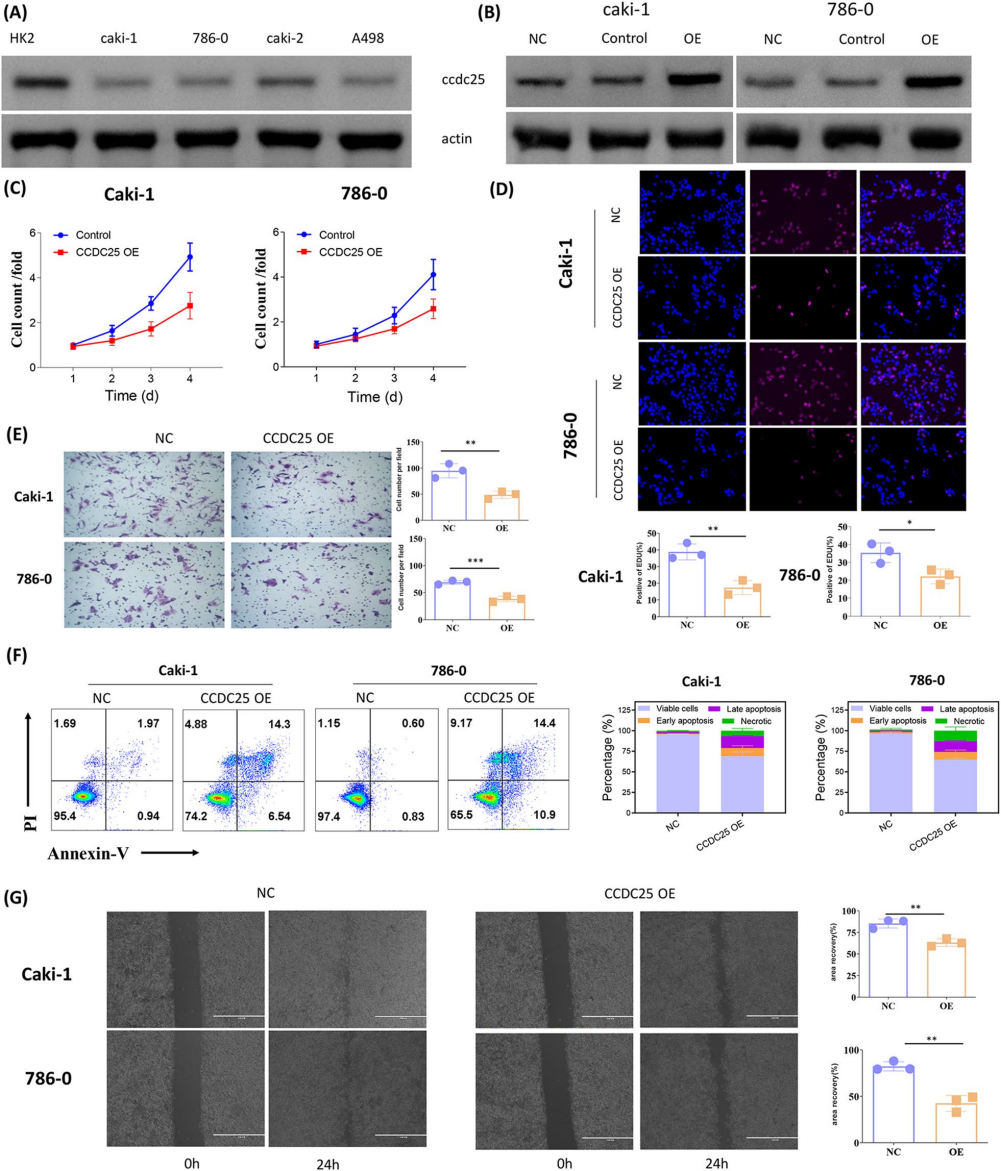
CCDC25 reduced the proliferation and migration of ccRCC in vitro. **A** CCDC25 protein levels in normal renal cell line (HK2) and ccRCC cell lines (Caki-1, 786–0, A498 and Caki-2) measured by western blotting. **B** Overexpression efficiencies of CCDC25 in Caki-1 and 786–0 cells was detected by Western blot analysis. **C** CCK-8 assays were used to detect the proliferation of Caki-1 and 786–0 cells after overexpression CCDC25 (TWO-WAY anova, * P < 0.05). **D** EdU assays and the relative quantitative results of cell proliferation are shown in histogram form (*p < 0.05, **p < 0.01). **E** Representative images and bar graphs of Transwell migration assays of Caki-1 and 786–0 cells after overexpression CCDC25 (**p < 0.01, ***p < 0.001,). **F** Apoptosis assays and the relative quantitative results. **G** Representative images and bar graphs of wound healing assays of Caki-1 and 786–0 cells after overexpression CCDC25 (**p < 0.01)

First, we examined the expression of CCDC25 in several renal cancer cell lines and a normal renal cell line (HK2) using Western blot analysis. The results showed that the expression of CCDC25 in the normal renal cell line HK2 was significantly higher than in the other renal cancer cell lines (Fig. 2A). By employing lentiviral transduction, we established stable cell lines of Caki-1 and 786–0 cells overexpressing CCDC25, which we will use for further research. We assessed the proliferation of ccRCC (clear cell renal cell carcinoma) cell lines under conditions of CCDC25 overexpression using the CCK8 assay. The results showed that the proliferation of both cell lines was significantly reduced upon overexpression of CCDC25 (Fig. 2C). EDU staining results revealed a notable decrease in EDU-positive cells following CCDC25 overexpression, further confirming that CCDC25 overexpression inhibits the proliferative capacity of tumor cells (Fig. 2D). Transwell assay results indicated that the migration ability of tumor cells markedly declined upon overexpression of CCDC25 (Fig. 2E). Apoptosis experiments showed that overexpression of CCDC25 promoted apoptosis of renal carcinoma cell lines (Fig. 2F). Scratch wound healing assays further corroborated this inhibitory effect on cell migration. Thus, these findings confirm that CCDC25 may exert an inhibitory effect on renal cancer cells (Fig. 2G).

### CCDC25 reduced Hippo pathway activation

**Fig. 3:**
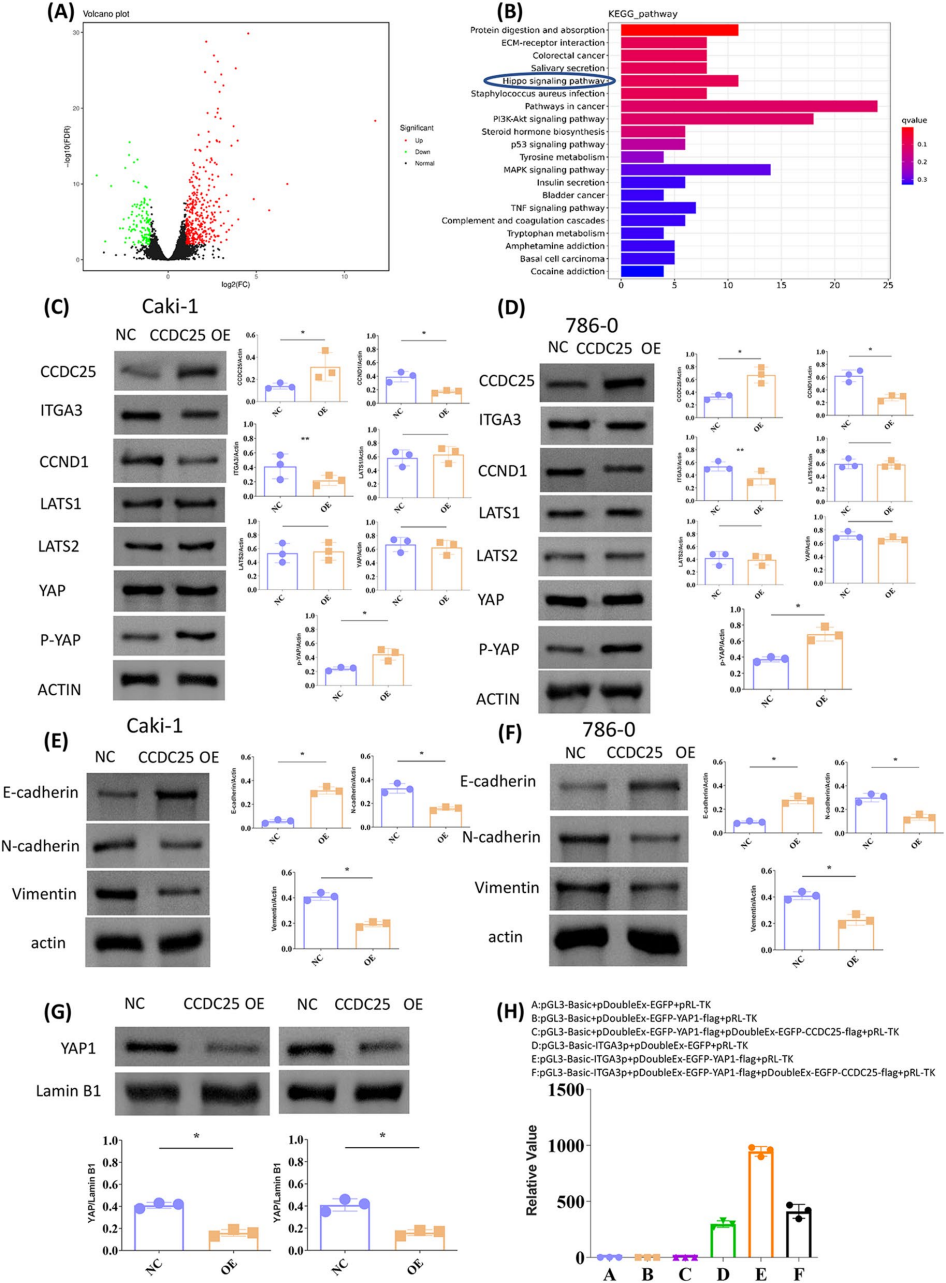
CCDC25 suppress Hippo pathway by promoting YAP phosphorylation and inhibiting its nuclear entry. **A** Volcano plot showing the differential genes in the 786–0-CCDC25 cell cluster. **B** KEGG plot showing pathway enrichment of differentially overexpressed genes in 786–0-CCDC25 cell cluster. **C**, **D** Representative immunoblots of biomarkers of proliferation and the Hippo pathway, using ACTIN as reference. Quantification of protein expression revealed that overexpressed CCDC25 notably inhibited the expression of downstream Hippo pathway proteins ITGA3 and CCND1, and elevated the expression levels of p-YAP. **E**, **F** Representative immunoblots of biomarkers of EMT pathway, using ACTIN as reference. Quantification of protein expression revealed that overexpressed CCDC25 notably elevated the expression levels of E-cadherin and inhibited the expression of N-cadherin and Vimentin. **G** Nuclear levels of YAP protein, using Lamin B1 as reference. Quantification of protein expression revealed that overexpressed CCDC25 notably reduced nuclear levels of YAP protein. **H** Dual-luciferase activity assay on the ITGA3 promoter, YAP and CCDC25. Data are presented as mean ± SD. *p < 0.05, **p < 0.01, ***p < 0.001, ns, no significance. Data are representative of three independent experiments in each group

We conducted RNA transcriptome sequencing analysis on 786–0 cells overexpressing CCDC25 and normal 786–0 cells to explore the potential mechanism of CCDC25 in renal cancer. Analysis revealed 478 differentially expressed genes, of which 355 were upregulated, and 123 were downregulated. KEGG pathway analysis of these differential genes suggested that CCDC25 may affect protein digestion and absorption, ECM-receptor interaction and the Hippo pathway, among others. Based on the ranking of q-values, we chose the Hippo pathway for further investigation (Fig. 3A, B).

**Fig. 4:**
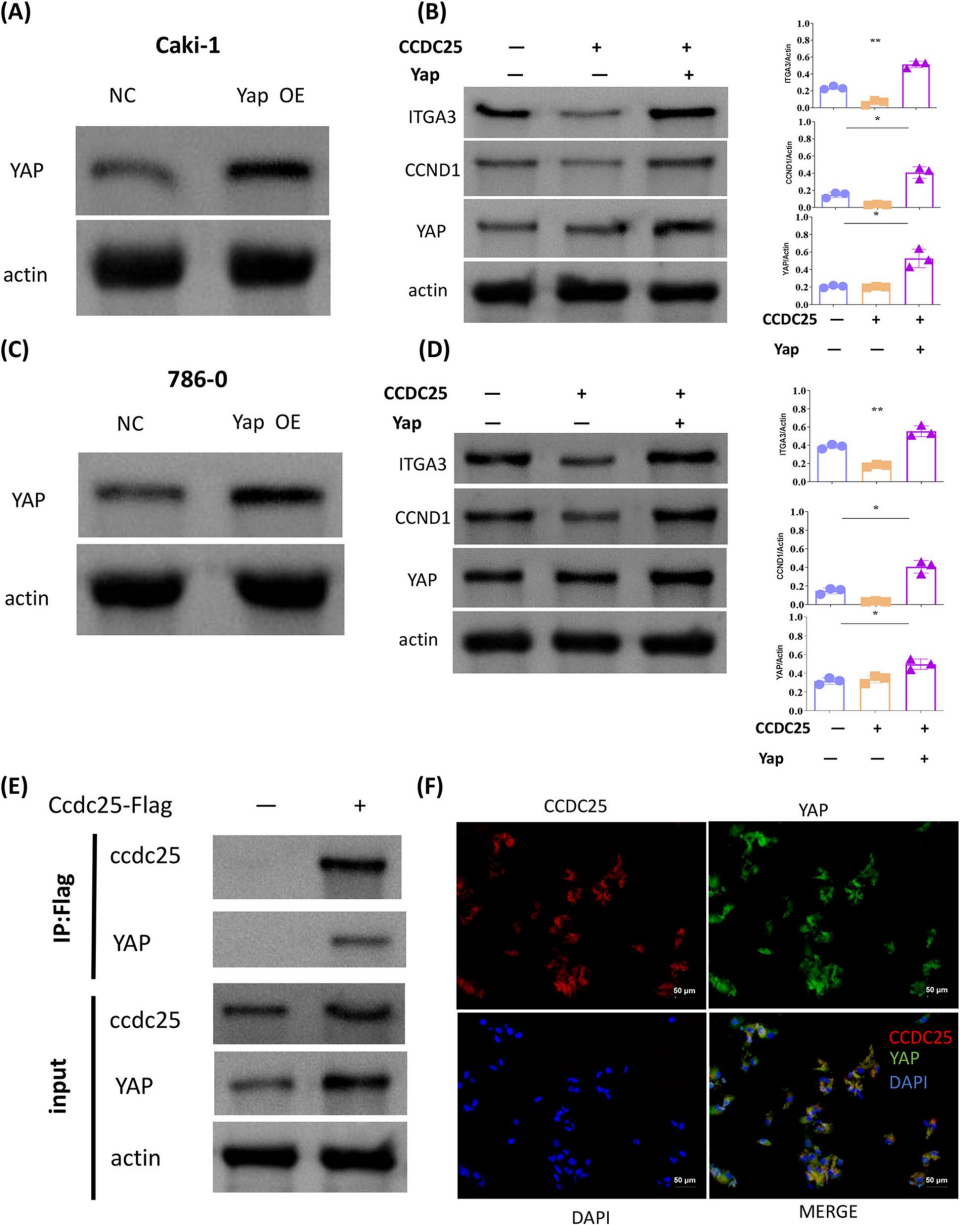
CCDC25 affects the Hippo pathway through YAP. **A** Overexpression efficiencies of YAP in Caki-1 and cells was detected by Western blot analysis. **C** Overexpression efficiencies of YAP in 786–0 and cells was detected by Western blot analysis. **B**, **D** Representative immunoblots of biomarkers of the Hippo pathway, using ACTIN as reference. Quantification of protein expression revealed that overexpression of YAP reversed the reduction of ITGA3 and CCND1 caused by CCDC25 overexpression. **E** 293 T cell lysates were prepared by weak RIPA lysis and co-immunoprecipitated by CCDC25 antibody with IgG as negative control. The result showed that YAP interacts with CCDC25. **F** Sub-localizations of YAP and CCDC25 were analyzed using immunofluorescence. Laser Confocal Microscopy photos showed that YAP and CCDC25 were co-localization both in the nuclei and cellular matrix. The scale bars represent 50 μm

We first evaluated the impact of CCDC25 overexpression on key proteins and downstream proteins in the Hippo pathway using Western blot (WB) analysis. The results showed that overexpression of CCDC25 notably inhibited the expression of downstream Hippo pathway proteins ITGA3 and CCND1 but did not affect the expression of the key protein YAP or the proteins LATS1/LATS2, which influence YAP phosphorylation. However, it did impact the phosphorylation of YAP (Fig. 3C, D). As ITGA3 is a crucial protein regulating tumor EMT (epithelial-mesenchymal transition), we further confirmed that overexpression of CCDC25 suppresses EMT in ccRCC cells (Fig. 3E, F). Relevant literature indicates that YAP promotes the transcriptional activation of downstream genes, such as ITGA3, by entering the nucleus, while phosphorylated YAP cannot enter the nucleus to exert its effect. As overexpression of CCDC25 promotes YAP phosphorylation, our subsequent results showed that the nuclear levels of YAP protein were significantly reduced under CCDC25 overexpression (Fig. 4G). Since YAP needs to enter the nucleus to activate its transcriptional promoting function, we wanted to further verify whether CCDC25's inhibition of yap into the nucleus would affect its transcriptional function. Therefore, a luciferase activity assay on the ITGA3 promoter confirmed that YAP can enhance the promoter activity of ITGA3 and that CCDC25 inhibits this effect. Therefore, CCDC25 may influence the activation of downstream genes by promoting YAP phosphorylation and inhibiting its nuclear entry (Fig. 4H).

### CCDC25 affects the Hippo pathway through YAP

We first overexpressed YAP through plasmid transfection, to verify whether CCDC25 affects renal cancer cells through YAP protein. The results showed that overexpression of YAP reversed the reduction of ITGA3 and CCND1 caused by CCDC25 overexpression (Fig. 4A–D). Further co-immunoprecipitation (co-IP) results revealed an interaction between CCDC25 and YAP proteins. Immunofluorescence colocalization further confirmed this finding (Fig. 4E, F).

### CCDC25 affects the hippo pathway by promoting YAP phosphorylation through LATS1

**Fig. 5:**
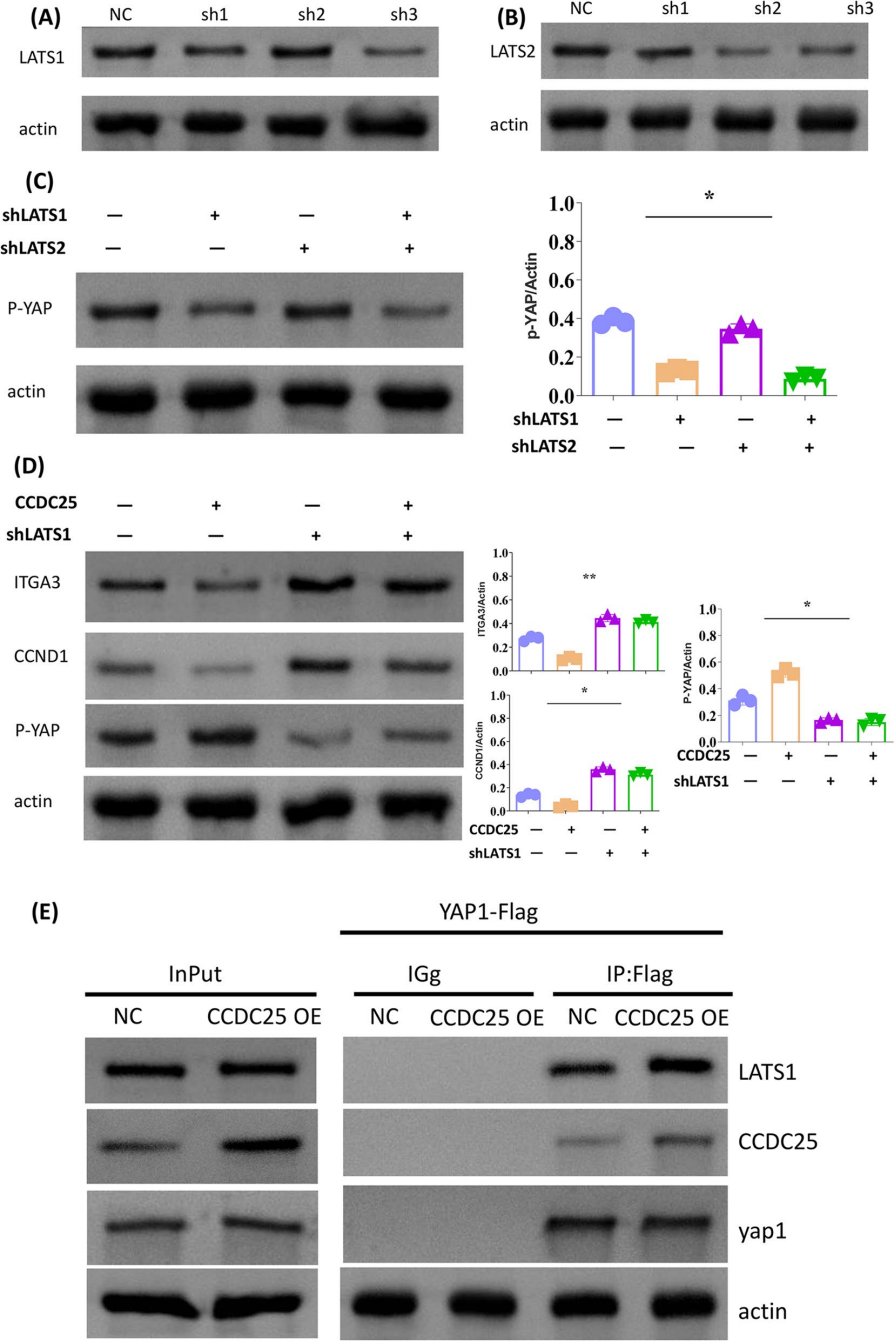
CCDC25 affects the hippo pathway by promoting YAP phosphorylation through LATS1. **A** Knockdown efficiencies of LAST1 in 786-0 cells was detected by Western blot analysis. **B** Knockdown efficiencies of LAST2 in 786-0 cells was detected by Western blot analysis. **C** YAP phosphorylation was detected by Western blot analysis, using ACTIN as reference. Quantification of protein expression revealed that knocking down LATS1 inhibited YAP phosphorylation, while the effect of LATS2 was relatively low. **D** Representative immunoblots of biomarkers of the Hippo pathway, using ACTIN as reference. Quantification of protein expression revealed that knockdown of LATS1 significant attenuated the increase of YAP phosphorylation and reduce of ITGA3 and CCND1 expression caused by CCDC25 overexpression. **E** 293 T cell lysates were prepared by weak RIPA lysis and co-immunoprecipitated by YAP1-Flag with IgG as negative control. The result showed that YAP interacts with CCDC25 and LATS1 and overexpression of CCDC25 promotes the binding of LATS1 to YAP.

**Fig. 6:**
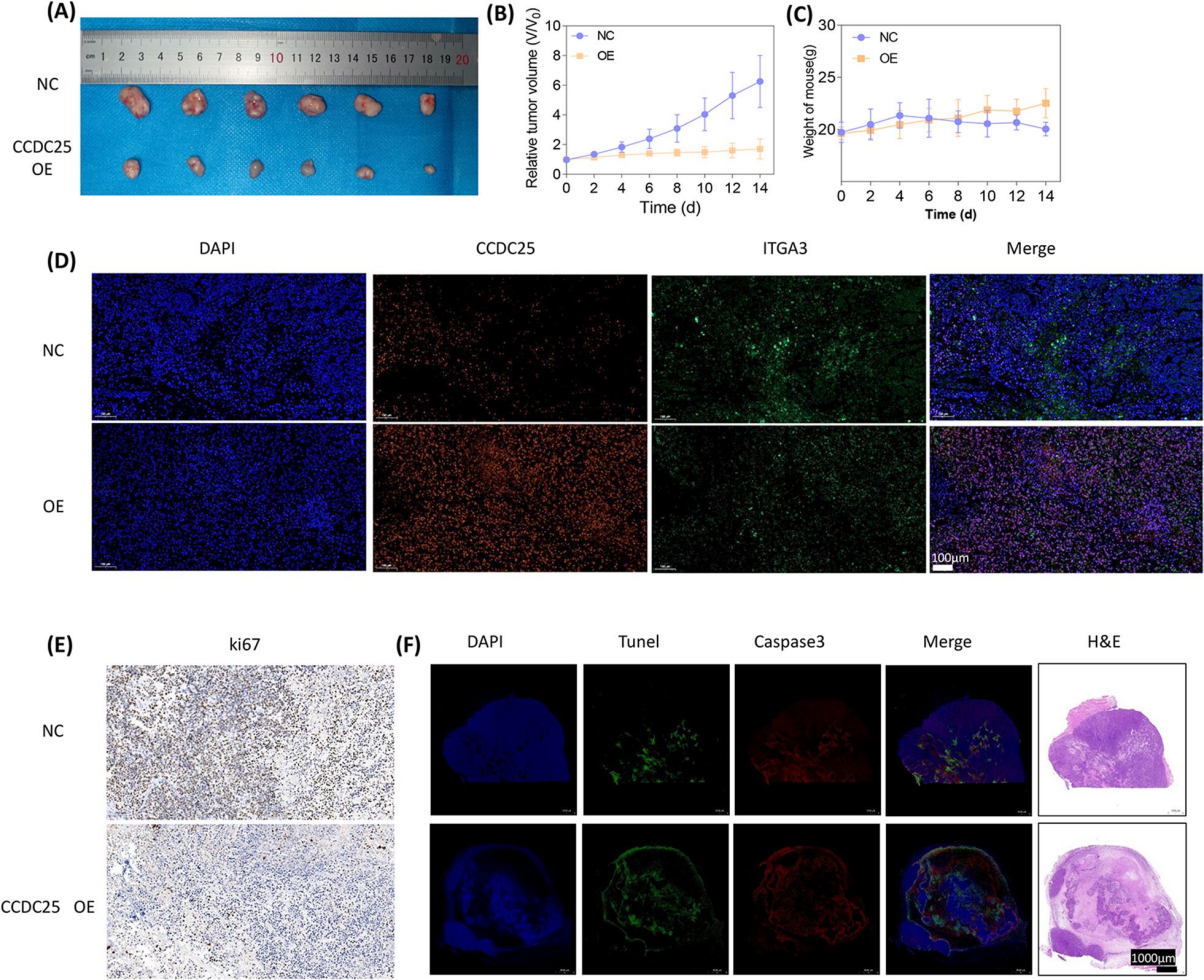
CCDC25 inhibits tumor growth and promotes apoptosis **A** Images of xenograft-transplanted nude mouse models (n = 6) and dissected tumor 14 days after injection with overexpressed CCDC25 786–0 cells and their corresponding NC cells. **B** Xenograft tumor growth volume curves of OE-CCDC25 cells and their corresponding NC cells. **C** Xenograft tumor weight curves of OE-CCDC25 cells and their corresponding NC cells. **D** Representative fluorescence images of Xenograft tumor labeled by DAPI, CCDC25, ITGA3. Scar bar, 100 μm. N = 6. **E** Xenograft tumor tissues were stained by IHC using ki-67 antibodies. The scale bar represents 100 μm. **F** H&E and fluorescence images of Xenograft tumor labeled by DAPI, Tunel, Caspase3

Since LATS1 and LATS2 primarily phosphorylate YAP, we sought to further understand how CCDC25 affects YAP phosphorylation. First, we used shRNA to suppress the expression of LATS1 and LATS2. Western blot (WB) results showed that in 786–0 cells, knocking down LATS1 inhibited YAP phosphorylation, while the effect of LATS2 was relatively low, which confirmed that LATS1 plays a primary role in YAP phosphorylation in 786–0 cells (Fig. 5A–C). Furthermore, under LATS1 knockdown conditions, the increased YAP phosphorylation and reduced ITGA3 and CCND1 expression caused by CCDC25 overexpression were significantly attenuated (Fig. 6D). Finally, through co-immunoprecipitation (COIP), we further verified that overexpression of CCDC25 promotes the binding of LATS1 to YAP (Fig. 6E). Therefore, CCDC25 may promote YAP phosphorylation by facilitating the interaction between LATS1 and YAP, thereby inhibiting the activation of the HIPPO pathway.

### CCDC25 inhibits tumor growth and promotes apoptosis

In vivo, we further verified the effects of CCDC25. We found that overexpression of CCDC25 significantly inhibited the growth of mouse xenograft tumors (Fig. 6A, B). Subsequent tissue section analysis of the harvested tumors revealed that in the CCDC25-overexpressing group, the expression of ITGA3 was significantly reduced (Fig. 6C), and the number of Ki67-positive cells was also significantly decreased. Moreover, it was observed that when CCDC25 was overexpressed, the expression of caspase-3 in the tumors and TUNEL staining were markedly increased (Fig. 6E, F). CCDC25 attenuates tumor growth and promotes apoptosis within the tumor tissue. Thus, CCDC25 also functions to suppress the growth of renal clear cell carcinoma in vivo.

## Discussion

Our study reveals a novel role for CCDC25 in the pathogenesis and progression of clear cell renal cell carcinoma (ccRCC). Our comprehensive in vitro and in vivo analyses have shown that CCDC25 acts as a crucial regulator of ccRCC cell proliferation, migration, and apoptosis, and its low expression is associated with poor clinical outcomes in ccRCC patients.

As a member of the CCDC family, CCDC25 has been studied for its role in neutrophil extracellular traps (NETs). As a receptor for NETs, it may be associated with liver metastasis of tumors [16–18]. However, it may also have different regulatory mechanisms. Firstly, it is a gene on chromosome 8p, generally associated with tumor inhibition activity [19, 20]. Studies have shown that CCDC25 is censored in HCC patients with poor prognosis [20–22], and related studies have shown that when CCDC25 is overexpressed, it significantly inhibits the proliferation activity of neural stem cells [23], so it may have the function of inhibiting cell viability. In this study, we found that the CCDC25 expression is downregulated in ccRCC tumors compared to normal kidney tissues and the expression of CCDC25 is inversely correlated with tumor stage, and patients with distant metastases exhibited significantly lower levels of CCDC25 expression compared to those without metastases. The survival analysis further corroborated this trend, which indicated that ccRCC patients with high CCDC25 expression have a significantly improved overall survival rate. The overexpression of CCDC25 reduced the proliferation and migration of renal cancer cells while promoting apoptosis. These effects were further validated in vivo mouse xenograft model.

In exploring the molecular mechanisms underlying the effects of CCDC25 on ccRCC, our RNA transcriptome sequencing analysis identified the Hippo pathway as one of the key pathways influenced by CCDC25 overexpression. The Hippo pathway is a well-known regulator of organ size and tissue homeostasis, and its dysregulation has been implicated in tumorigenesis [24–27]. YAP protein is the core effector molecule of hippo pathway. The activity of YAP is stringently controlled by the kinases MST1/2 and LATS1/2, which, upon phosphorylating YAP, confine it to the cytoplasm where it is targeted for degradation. Deactivation of these upstream kinases facilitates the movement of YAP into the nucleus of the cell, triggering the activation of genes downstream. In our study, we found that overexpression of CCDC25 inhibited the expression of downstream Hippo pathway proteins ITGA3 and CCND1. CCND1 is a common oncogene, while ITGA3 is a key gene that regulates epithelial-mesenchymal transformation [28–31]. Our further experimental results show that CCDC25 promotes YAP phosphorylation and reduces nuclear entry. Our study further revealed that CCDC25 promotes the binding of LATS1 to YAP, a crucial step in the phosphorylation of YAP. LATS1, as a key protein regulating YAP phosphorylation, inhibits YAP entry into the nucleus and further downstream transcriptional activation [32–35]. It often acts as a tumor suppressor, and its inhibitory effect on EMT has been confirmed in ccRCC [36, 37]. Interestingly, the knockdown of LATS1 significantly attenuated the increased YAP phosphorylation and reduced ITGA3 and CCND1 expression caused by CCDC25 overexpression. These findings indicate that CCDC25 may modulate the Hippo pathway by facilitating the interaction between LATS1 and YAP, thereby promoting YAP phosphorylation and inhibiting the activation of downstream genes. Our sequencing results show that CCDC25 may also affect PI3K and MAPK pathways, research has shown that the CCDC family gene CCDC134 can suppress the progression of gastric cancer by inhibiting the MAPK pathway [38]. Additionally, it has been reported that CCDC19 can inhibit both the PI3K/AKT and EGFR pathways [39, 40]. These findings suggest the potential significance of investigating the impact of CCDC25 on these pathways, indicating a promising area for further research to understand its role and effects more comprehensively.

In light of these considerations, future research should focus on delineating the molecular mechanisms underlying CCDC25's regulation of the Hippo pathway and its broader implications in RCC pathobiology. Additionally, the potential of CCDC25 as a prognostic marker warrants comprehensive validation in larger, multicentric cohorts. Ultimately, the development of CCDC25-targeted therapies could offer a promising addition to the current treatment landscape for ccRCC, especially for patients with advanced disease stages.

## Conclusion

Our findings underscore the importance of CCDC25 as a novel regulator of ccRCC progression. The downregulation of CCDC25 is associated with advanced tumor stage and poor prognosis in ccRCC patients. We have provided evidence that CCDC25 acts as a tumor suppressor by inhibiting cell proliferation, migration, and promoting apoptosis. Furthermore, our study has uncovered a novel mechanism by which CCDC25 modulates the Hippo pathway through interactions with LATS1 and YAP, revealing a potential therapeutic target for ccRCC. Overall, our findings provide new insights into the pathogenesis of ccRCC and pave the way for developing novel targeted therapies for this aggressive malignancy.

### Supplementary Information

**Additional file 1:** 1. Supplementary Methods. 2. Uncropped gel and blot images of western blot.

## Data Availability

The data used to support the findings of this study are available from the corresponding author upon request.
