# Supplementary material for: CCDC25 suppresses clear cell renal cell carcinoma progression by LATS1/YAP-mediated regulation of the hippo pathway
Source: Cancer Cell Int. 2024 Apr 3;24:124. doi: 10.1186/s12935-024-03318-0 (PMC10988808; doi:10.1186/s12935-024-03318-0)
Supplement: Supplementary file 1 — Additional file 1: 1. Supplementary Methods. 2. Uncropped gel and blot images of western blot. [file 12935_2024_3318_MOESM1_ESM.docx]

**Supplementary Information:**

1. Supplementary Methods

2. Uncropped gel and blot images of western blot

**Supplementary Experimental Materials**

**Cell culture**

Caki-1: McCoy's 5A＋10% FBS

Caki-2: McCoy's 5A＋10% FBS

786-o: RPMI-1640＋10% FBS

A498: DMEM＋10% FBS

HK2: DMEM＋10% FBS

293T: DMEM＋10% FBS

**Immunohistochemistry**

Tumors were first fixed and embedded in paraffin. Paraffin blocks were sectioned and deparaffinized with xylene, and the tissue sections were rehydrated in alcohol with decreasing concentrations and immersed in 3% H_2_O_2_ at room temperature for 10 min. Then, the sections were boiling for 30 min in 0.01 mol/L citrate buffer (pH = 6.0) for antigen retrieval. After that, the tissue sections were cooled to room temperature before being blocked in 10% BSA. Tissue samples were then incubated with primary antibodies at 4 °C overnight. After washing with PBS for 3 times. Finally, the tissue sections were counterstained with hematoxylin, dehydrated with ethanol and mounted with coverslips.

**Immunofluorescence**

The cells were grown on coverslips and washed three times with PBS. The cells were fixed with 4% paraformaldehyde at room temperature for 20 min and permeabilized with 0.1% Triton X-100 for 20 min. Following permeabilization, the cells were incubated with 5% BSA in PBS for 30 min at room temperature and then incubated with primary antibodies overnight at 4 °C. After three washes with PBS, the cells were incubated with secondary antibodies conjugated with Alexa Fluor-594 or -488 (1:200, Invitrogen) for 1h at room temperature. Finally, the nuclei were stained with 4′,6′-diamidino-2-phenylindole (DAPI) and the immunofluorescence images were obtained by confocal microscopy (Carl Zeiss, Germany).

**Western blot**

Whole cells were harvested and lysed in ice-cold RIPA buffer (Beyotime, P0013K) containing protease inhibitors cocktail (Roche Diagnostics, 05892970001) and phosphatase inhibitor cocktail (Roche Diagnostics, 04906845001). Cells lysates were centrifugated at 12,000 rpm for 20 min at 4 °C. The total protein concentration was measured by BCA protein assay kit (Thermo Fisher Scientific, 23225). Equal amounts of protein samples were separated by 10% SDS/PAGE, and transferred to nitrocellulose (NC) membranes (Pall Corporation). After blocking with 5% BSA, the membrane was incubated with the primary antibodies. After washing, the membranes were incubated with secondary antibodies. Signals were detected using Odyssey imaging system (Li-Cor Biosciences, NE) or using the Bio-Rad gel documentation system with the Super Signal West Dura Extended Duration Substrate (Thermo Fisher Scientific, 34076).

**Co-immunoprecipitation**

Cells were collected and lysed in ice-cold IP lysis buffer (Beyotime, P0013) and lysates were cleared using brief centrifugation. Then, lysates (approximately 100-400 µg total cellular protein) were transferred to a 1.5 ml microcentrifuge tube. 1 µg primary antibody was added and incubated for 1 hour at 4 °C. Then, 30 µl of protein A/G beads (Santa Cruz, sc-2002) were added and incubated at 4 °C on a rotating device overnight. Immunoprecipitates were collected by centrifugation at 2,500 rpm for 5 min at 4 °C and supernatant discarded carefully. Afterwards pellets were washed three times with the cold 1 x TBS, and then was resuspended in 50 µl of 2 x SDS loading buffer. The samples were boiled and subjected to Western Blot analysis.

**Nuclear Extraction**

The cells were washed with ice-cold PBS and then resuspended in buffer A (10 mM HEPES pH 7.9, 1.5 mM MgCl_2_, 10 mM KCl, 0.5 mM DTT, 0.15% NP40 and protease inhibitor cocktail). After 10 min on ice, the cells were centrifuged at 12,000 rpm and 4 °C for 30 sec. Remove the supernatant. The pellet was then resuspended with buffer B (10 mM Tris-HCL pH 7.6, 0.4 M NaCl, 2 mM MgCl_2_, 1 mM DTT, 0.5% NP40 and protease inhibitor cocktail). After three vigorous vortexing every 10 min, the homogenates were centrifuged at 12,000 rpm, 4 °C for 20 min. The resultant supernatants were then kept as nuclear fraction. Lamin B1 is marker for nuclear.

**Transfection efficiency diagram**


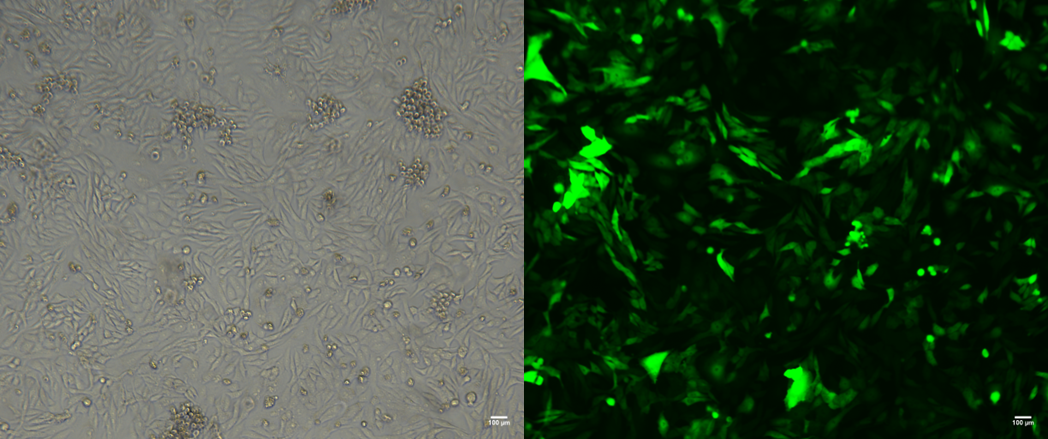


HK2 cell line growth curve after CCDC25 knockdown.


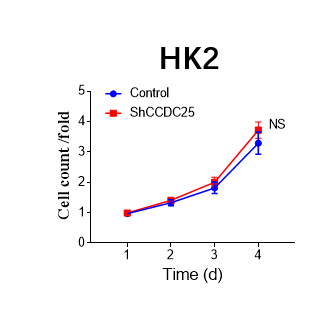


**Uncropped gel and blot images of western blot**

**
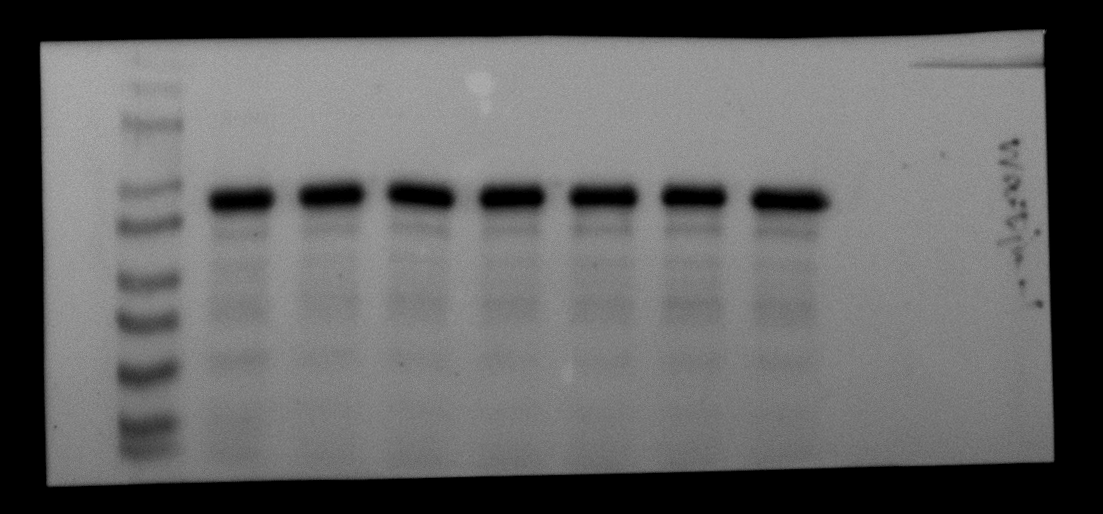
**

**Actin
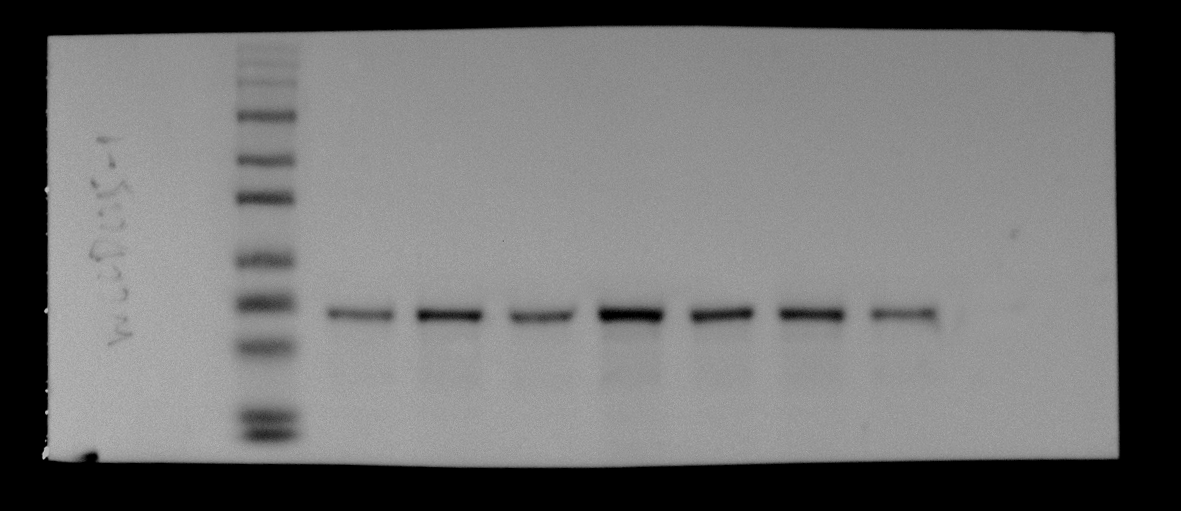
**

**CCDC25
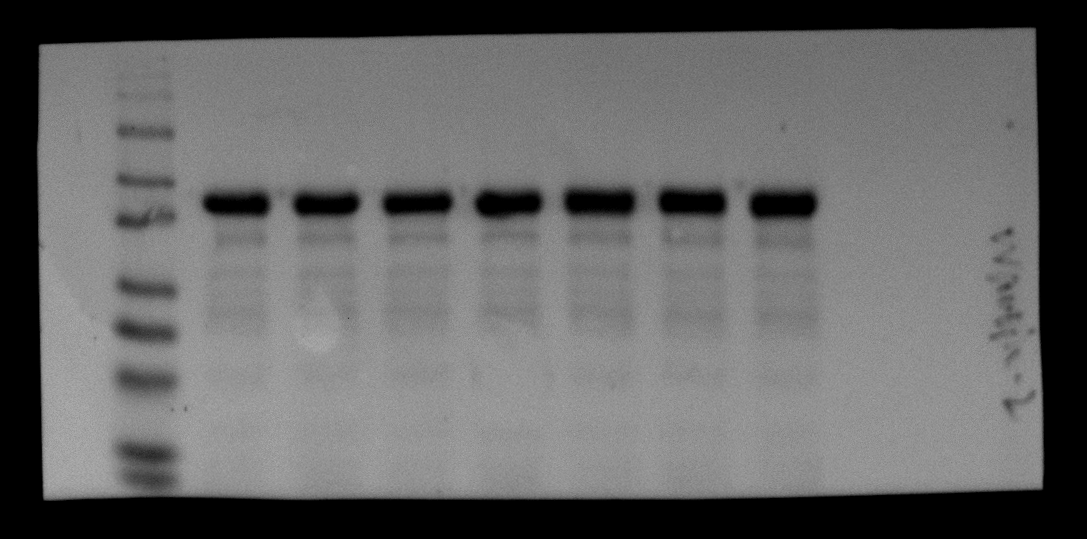
**

**ACTIN
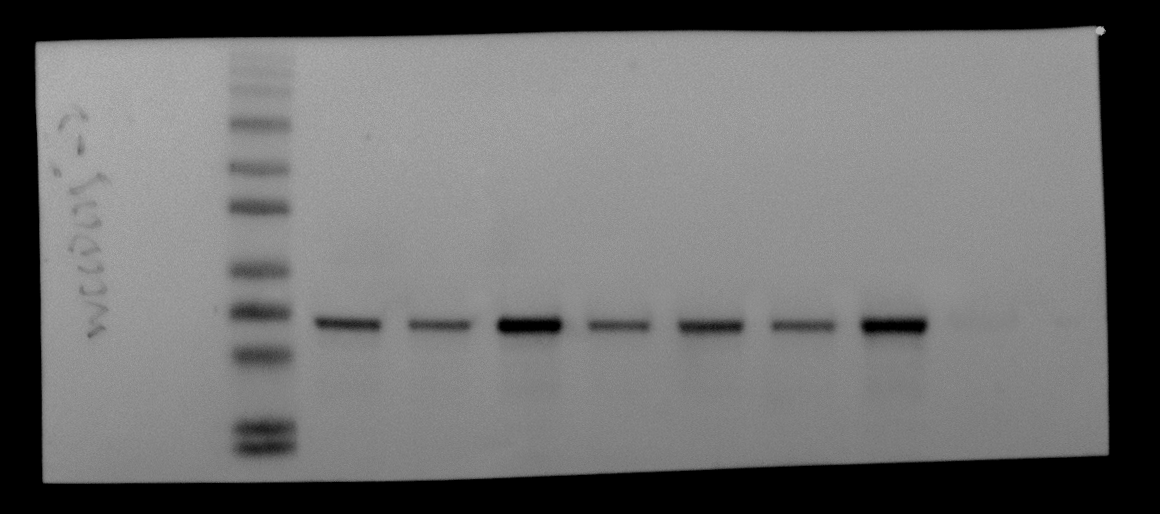
**

**CCDC25
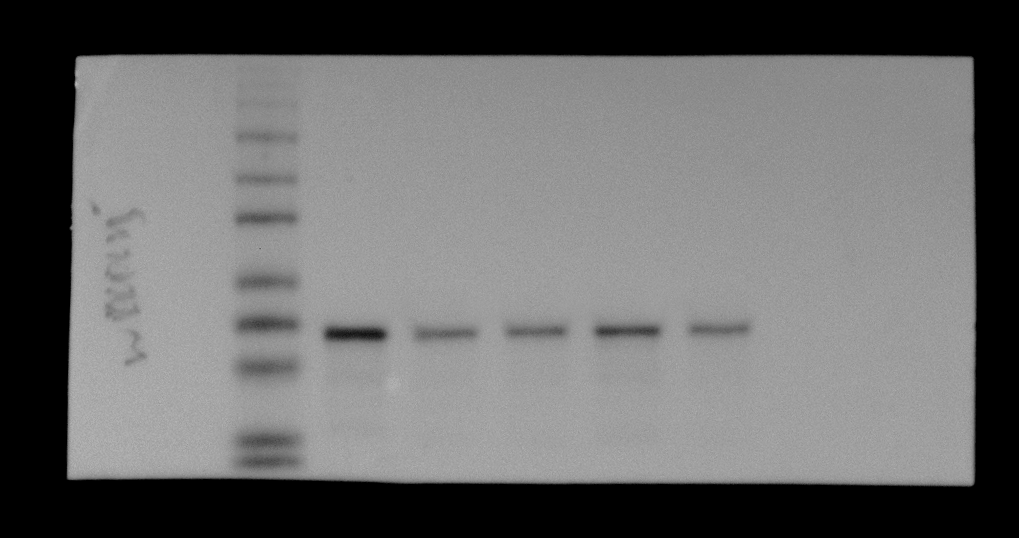
**

**CCDC25**

**
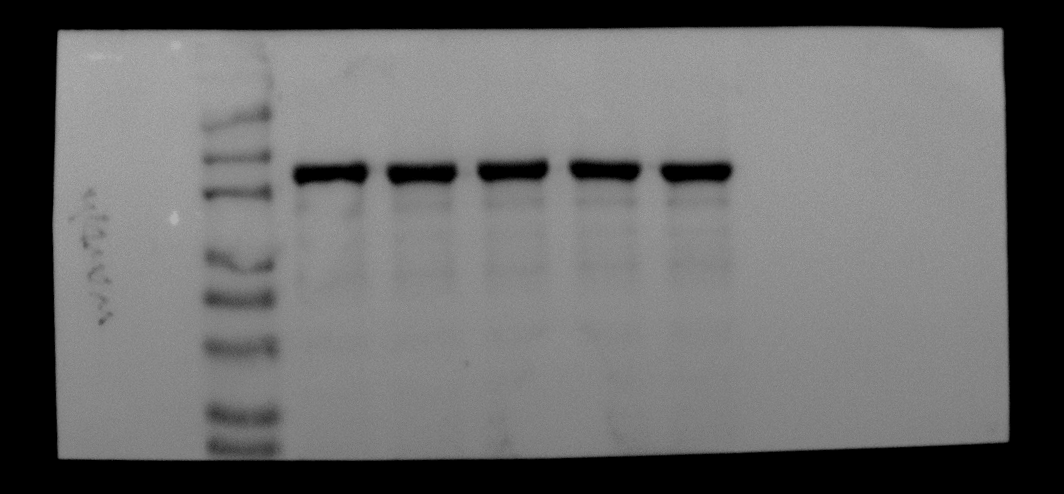
**

**ACTIN**

**
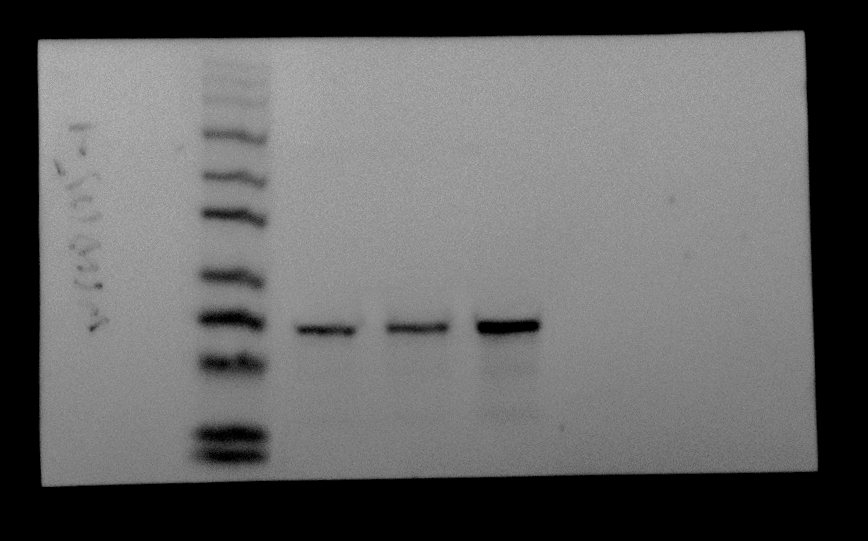
**

**CCDC25
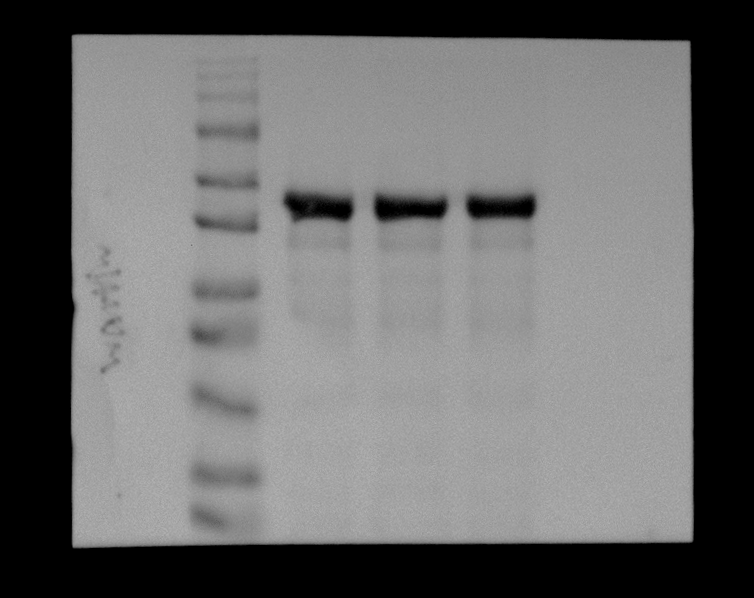
ACTIN**

**
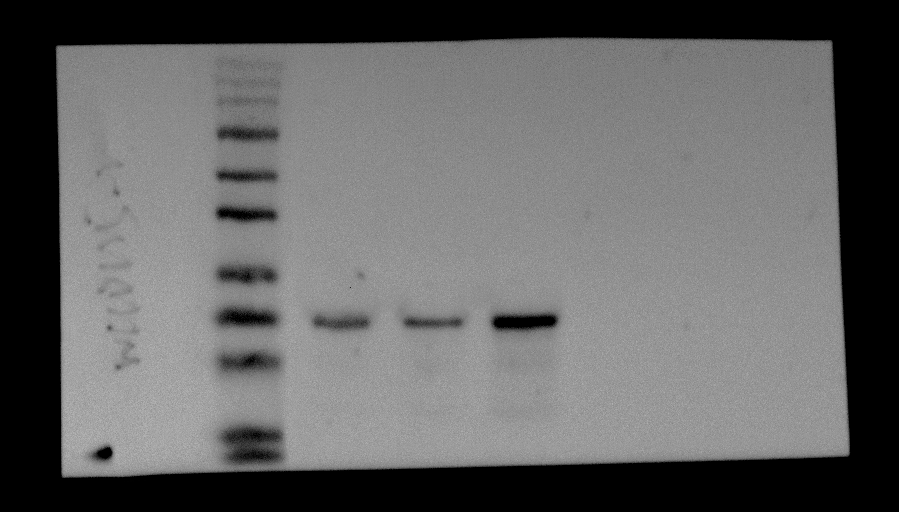
**

**CCDC25
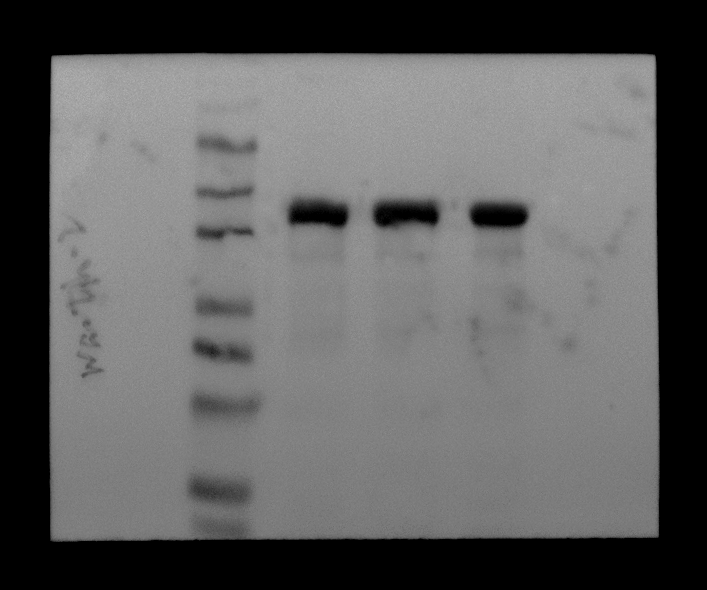
**

**ACTIN
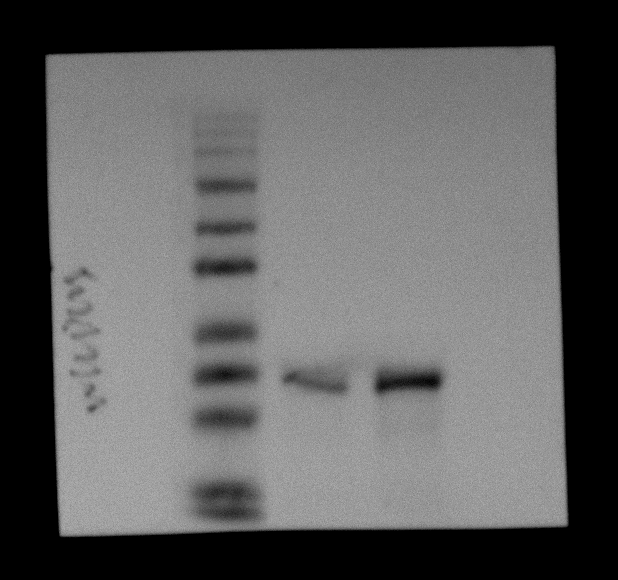
**

**CCDC25
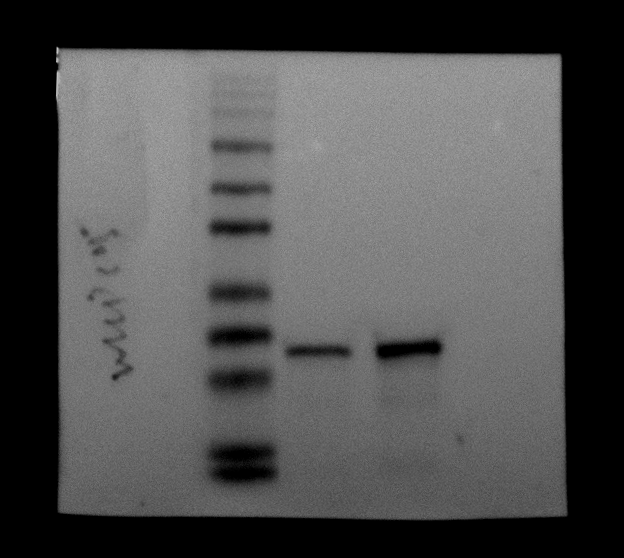
**

**CCDC25
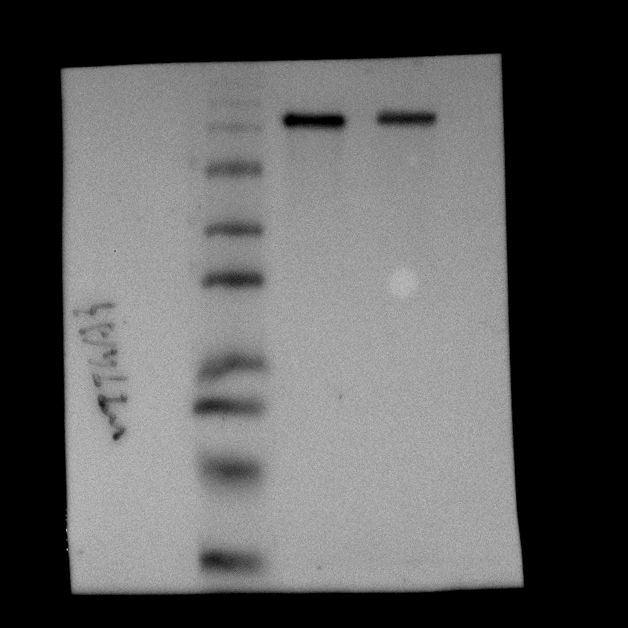
**

**ITGA3
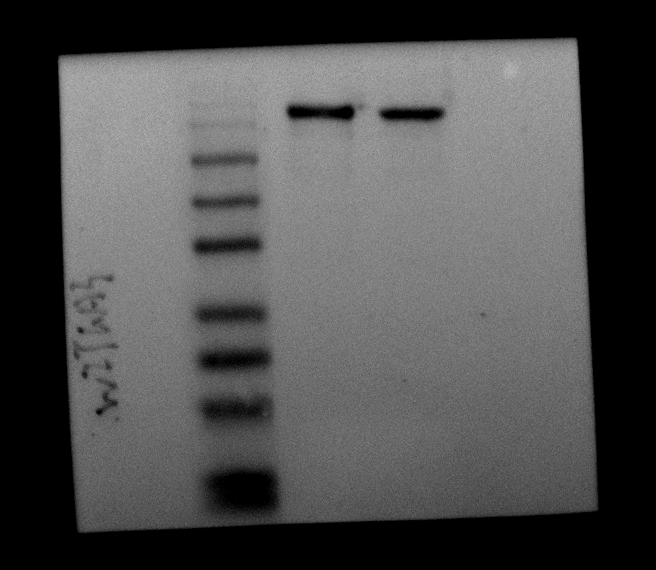
**

**ITGA3
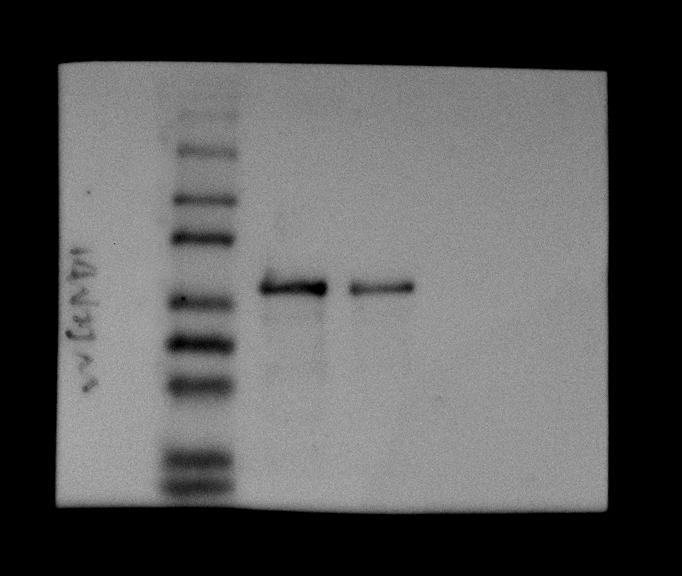
**

**CCND1**

**
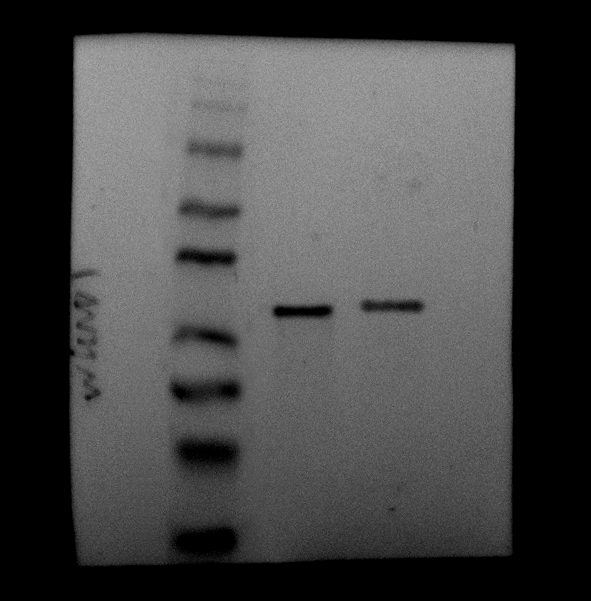
**

**CCND1**

**
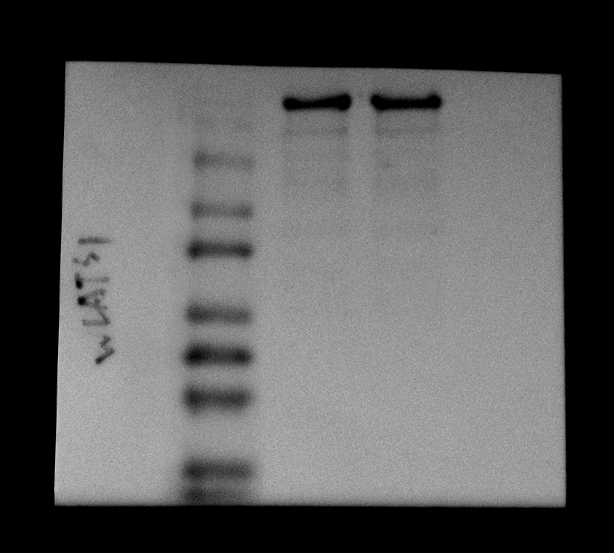
**

**LATS1**

**
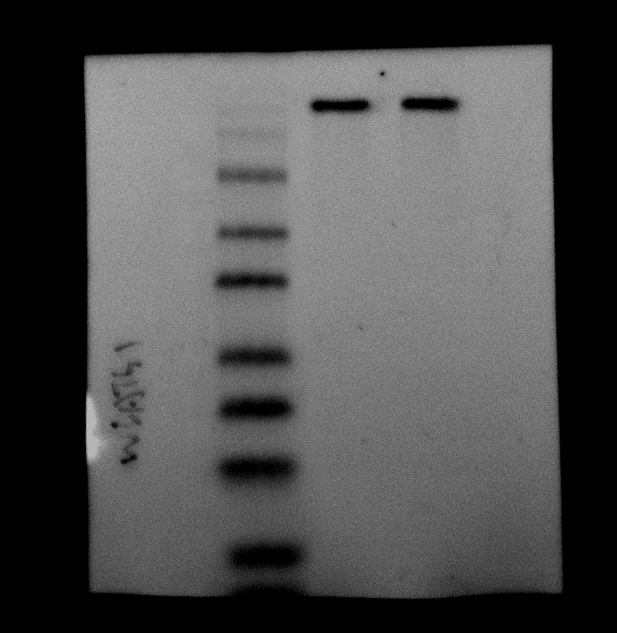
**

**LATS1**

**
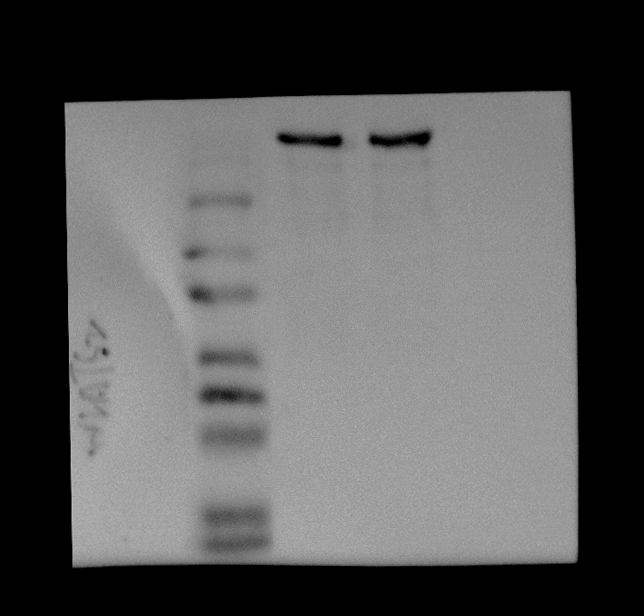
**

**LATS2**

**
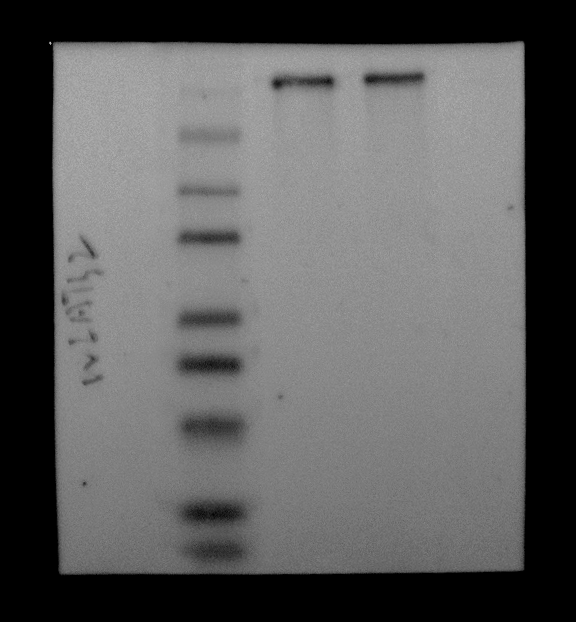
**

**LATS2**

**
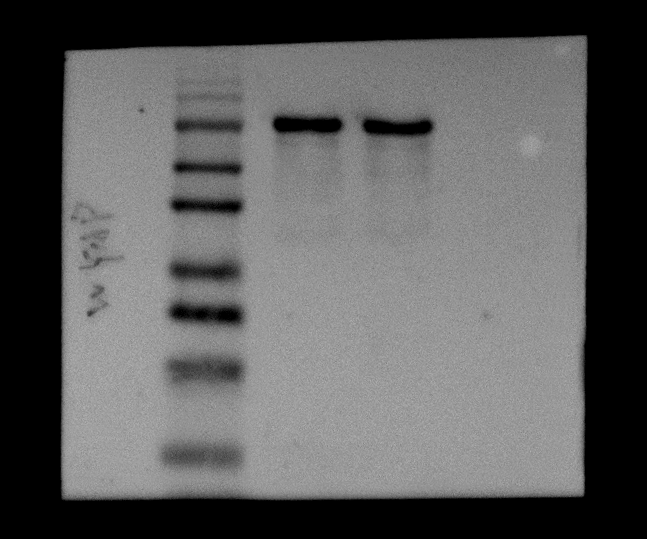
**

**YAP**

**
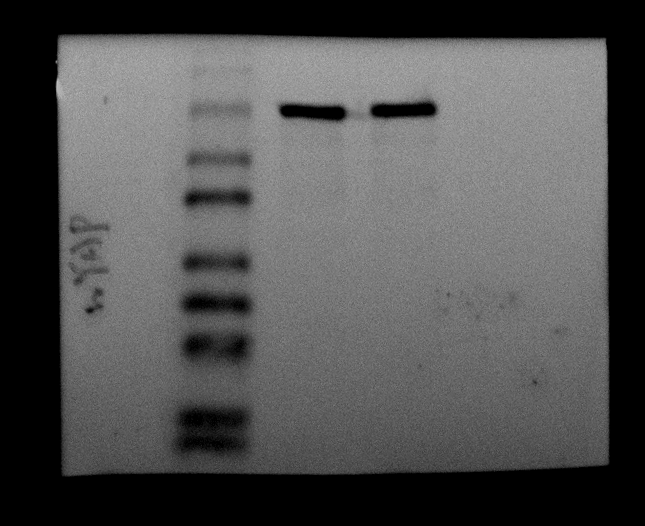
**

**YAP**

**
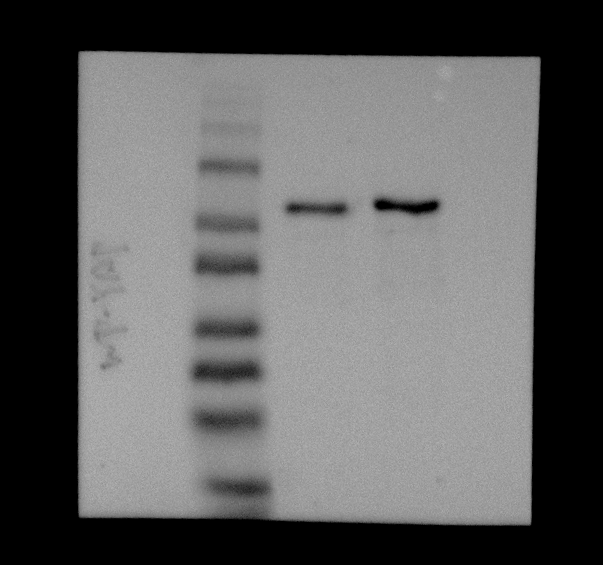
**

**P-YAP**

**
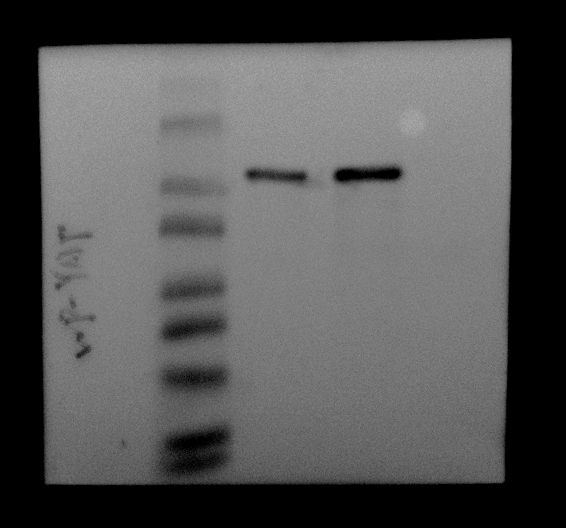
**

**P-YAP**

**
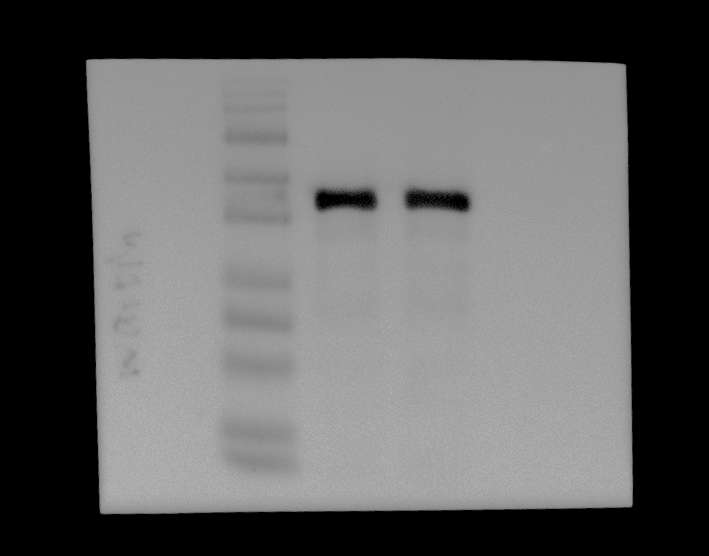
**

**ACTIN**

**
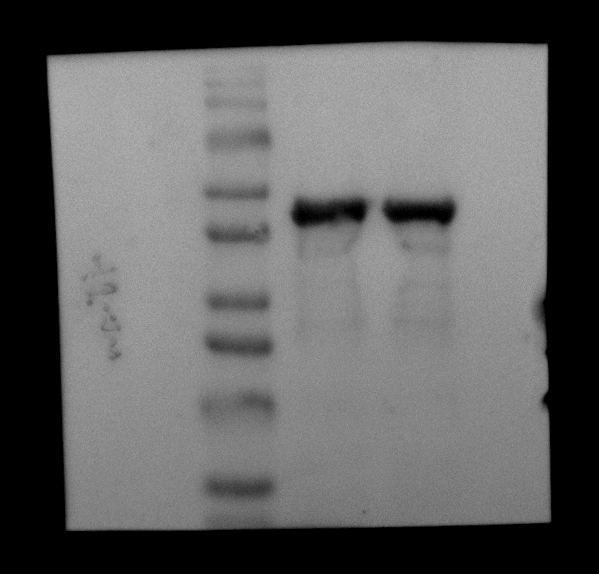
**

**ACTIN**

**
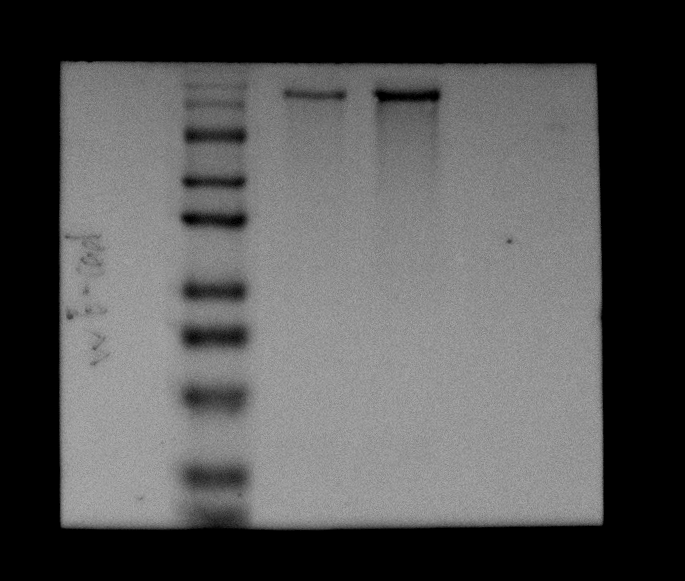
**

**E-CAD**

**
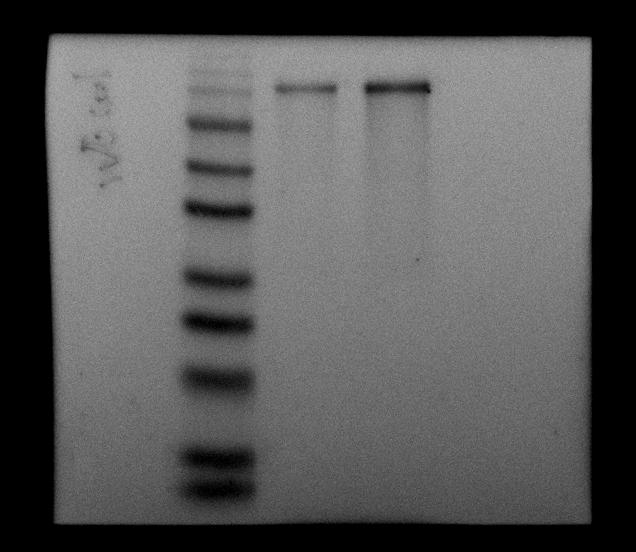
**

**E-CAD**

**
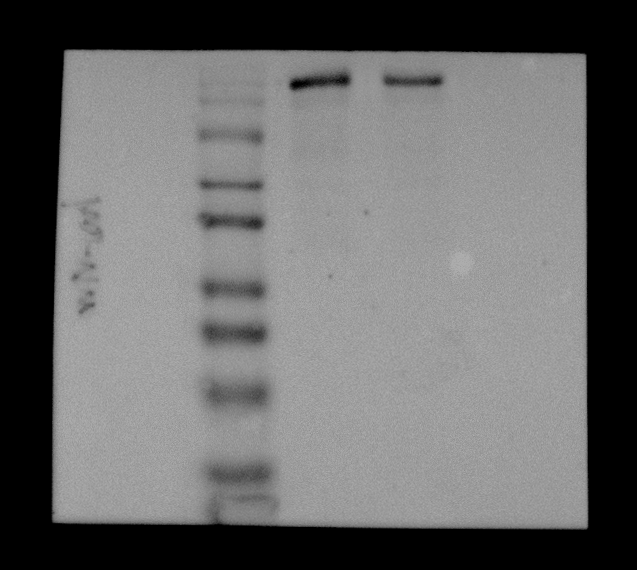
**

**N-CAD**

**
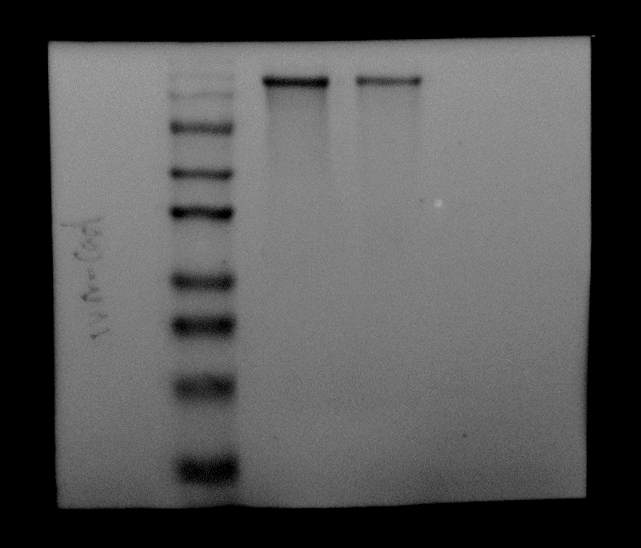
**

**N-CAD**

**
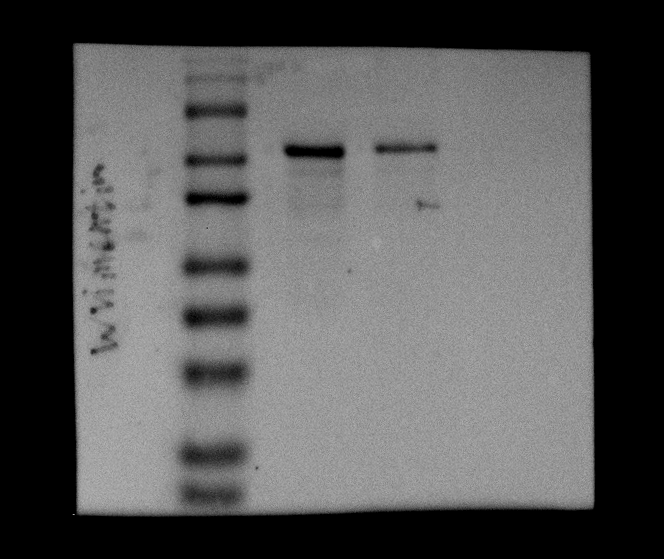
**

**VIMEN**

**
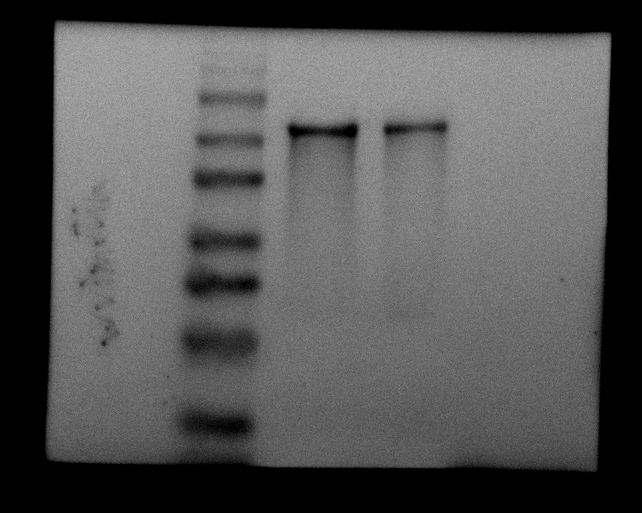
**

**VIMEN**

**
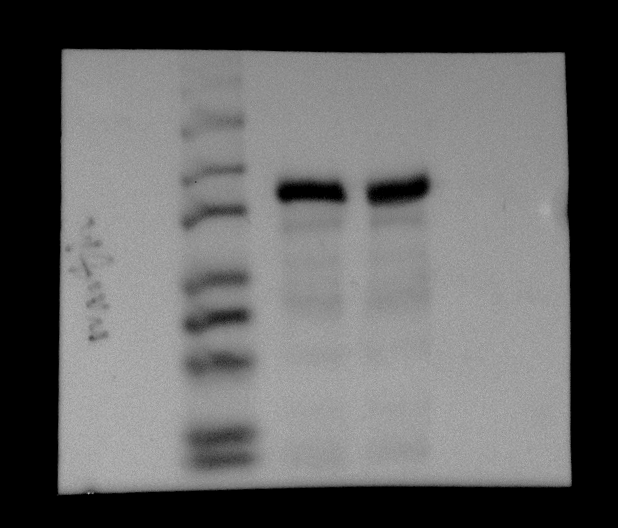
**

**ACTIN**

**
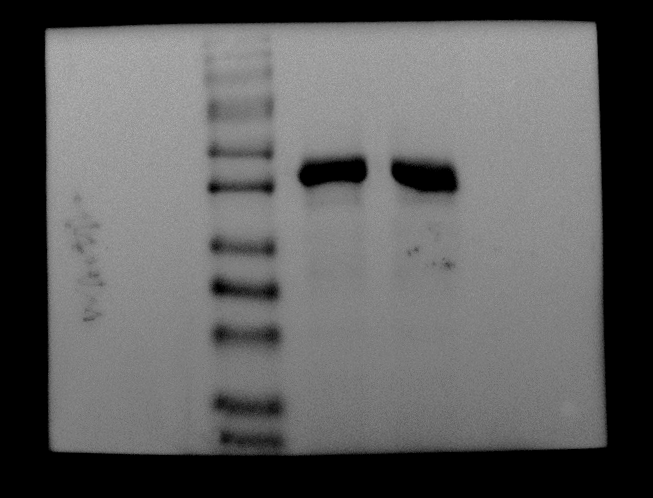
**

**ACTIN**

**
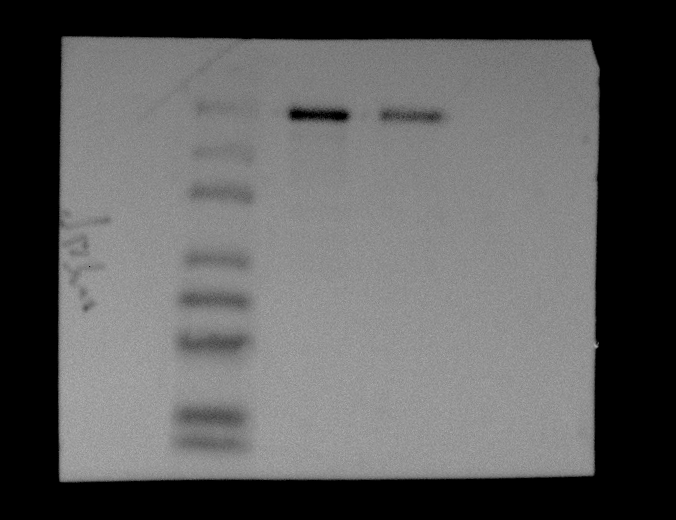
**

**YAP**

**
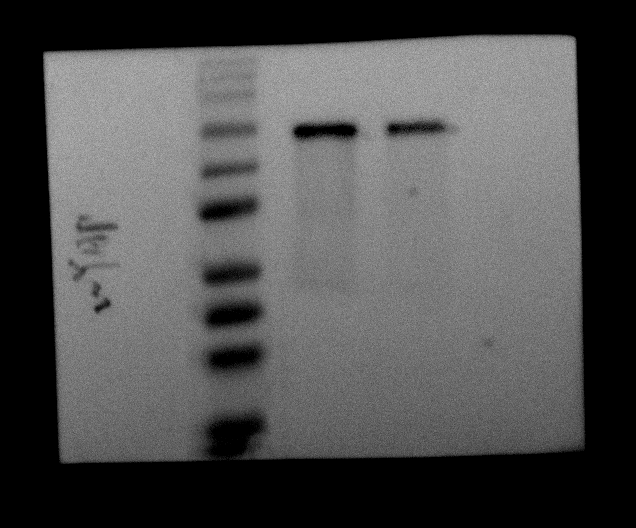
**

**YAP**

**
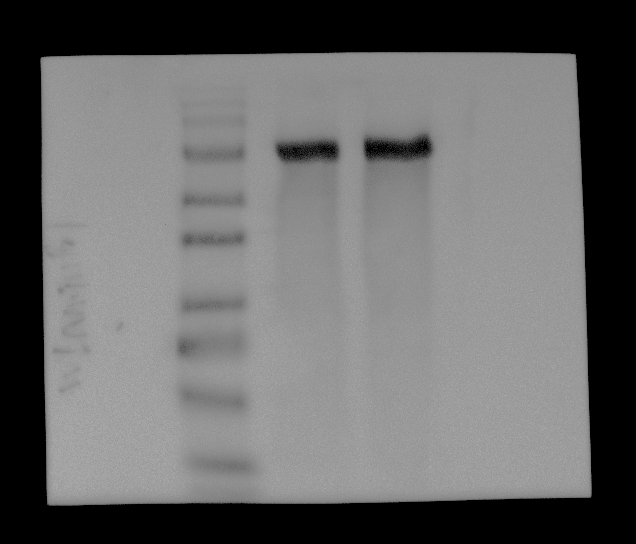
**

**LAMINB1
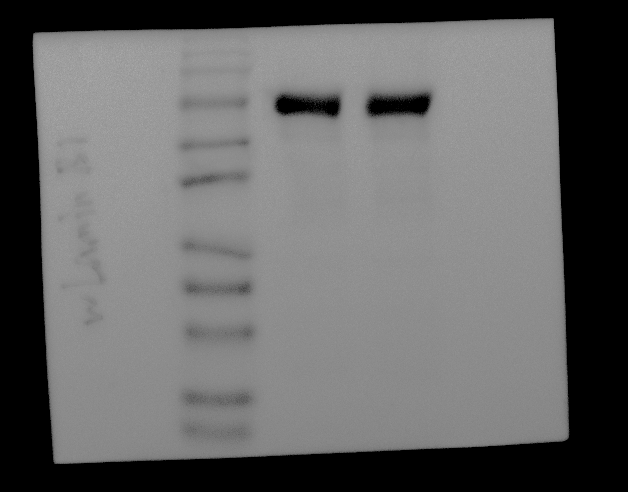
**

**LAMINB1**

**
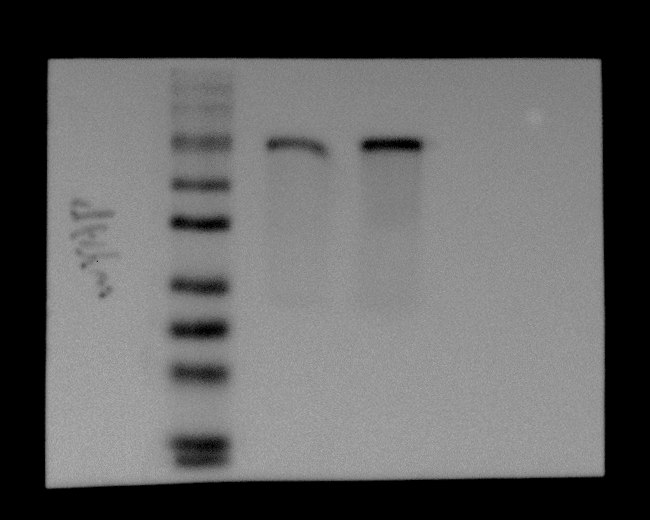
**

**YAP
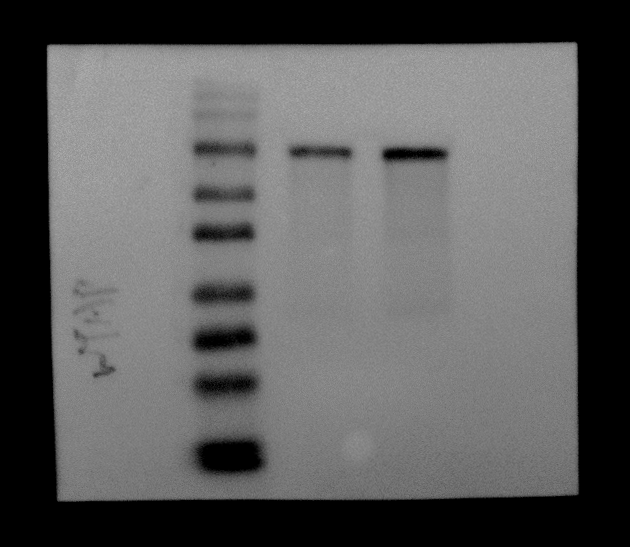
**

**YAP
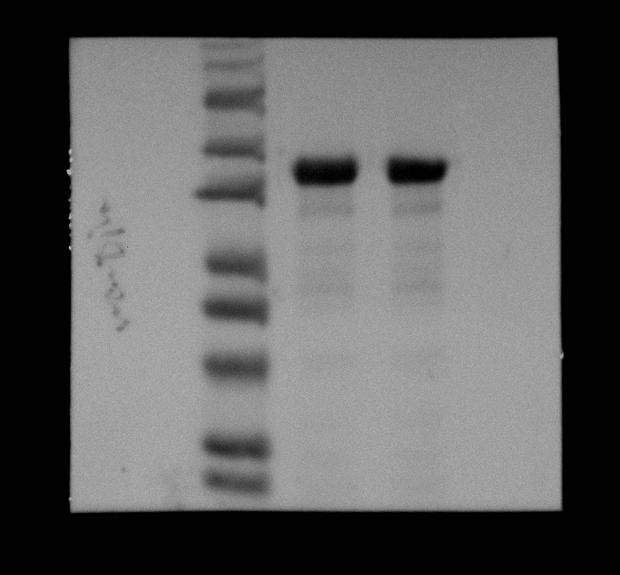
**

**ACTIN**

**
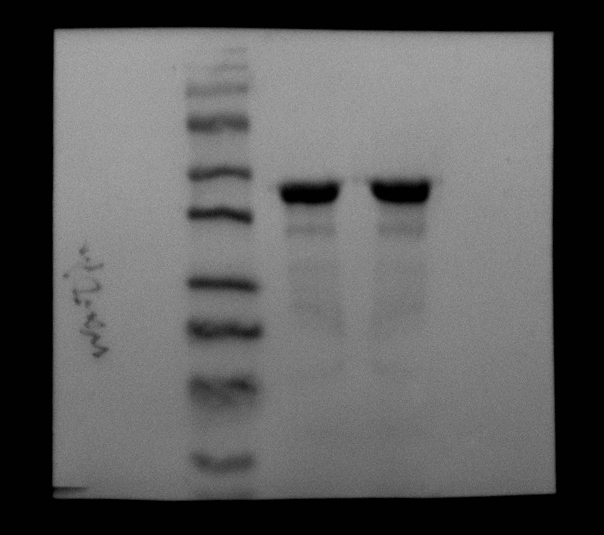
**

**ACTIN**

**
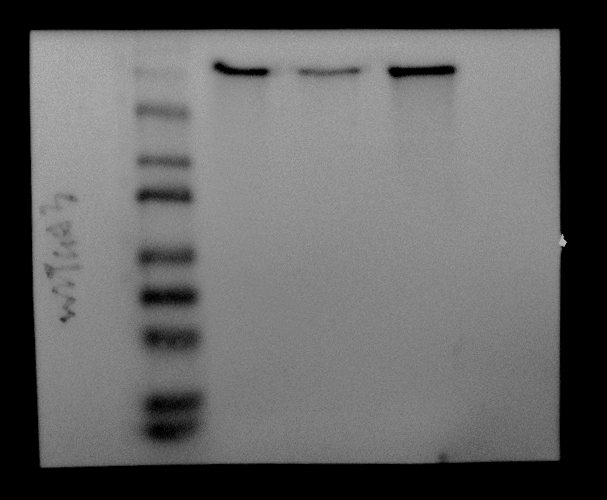
**

**ITGA3
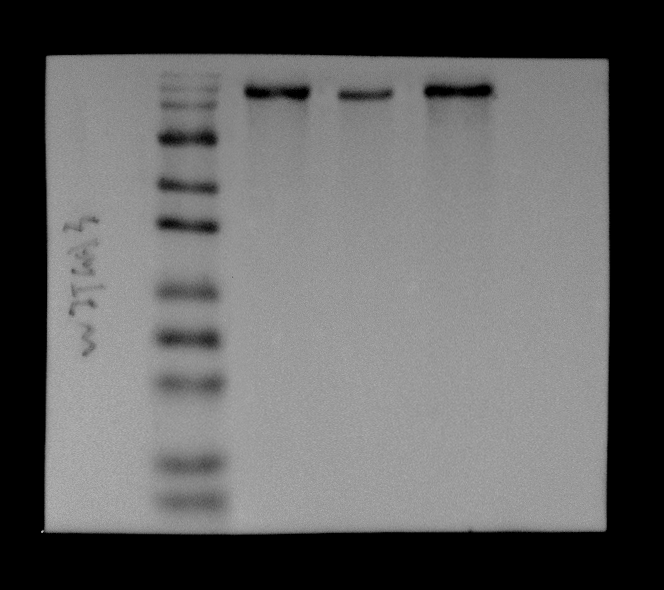
**

**ITGA3**

**
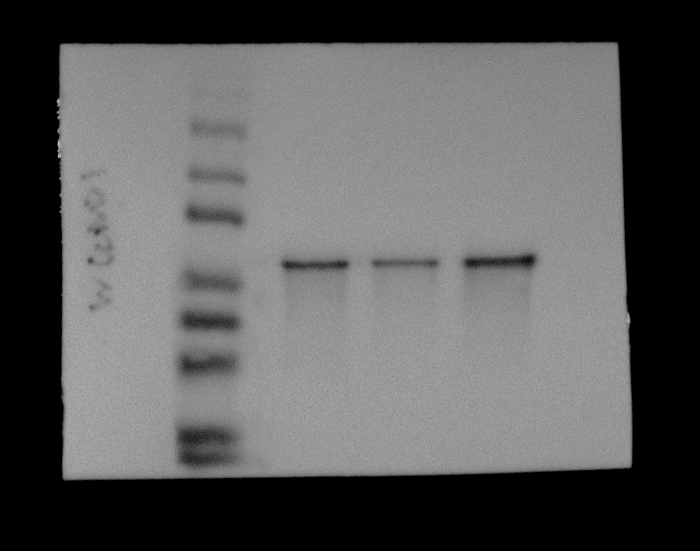
**

**CCND1
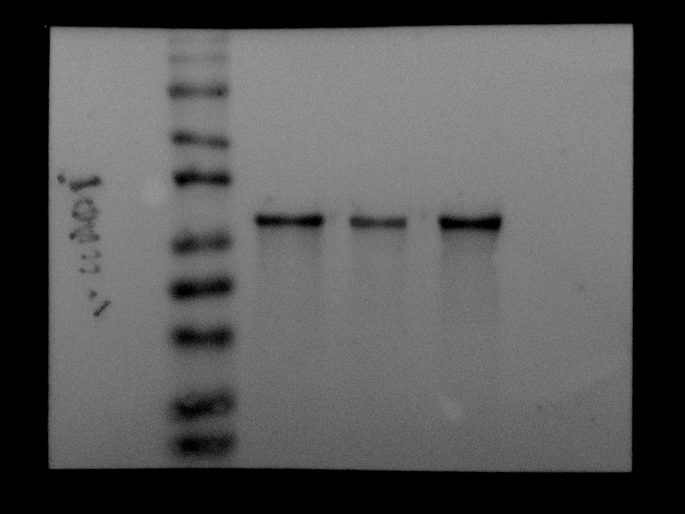
**

**CCND1**

**
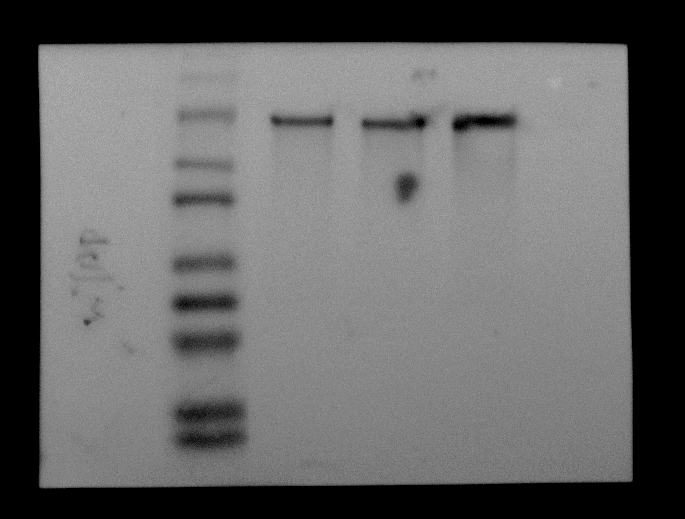
**

**YAP**

**
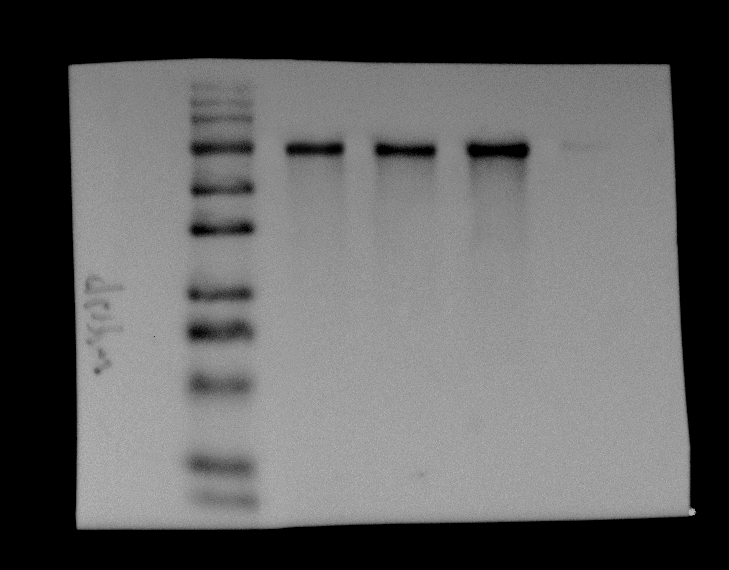
**

**YAP**

**
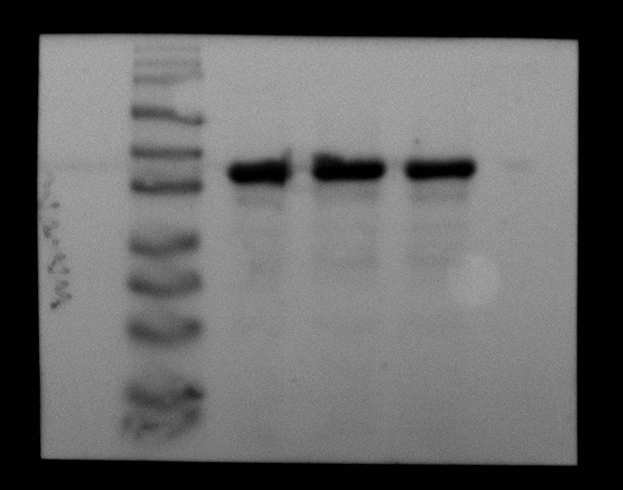
**

**ACTIN**

**
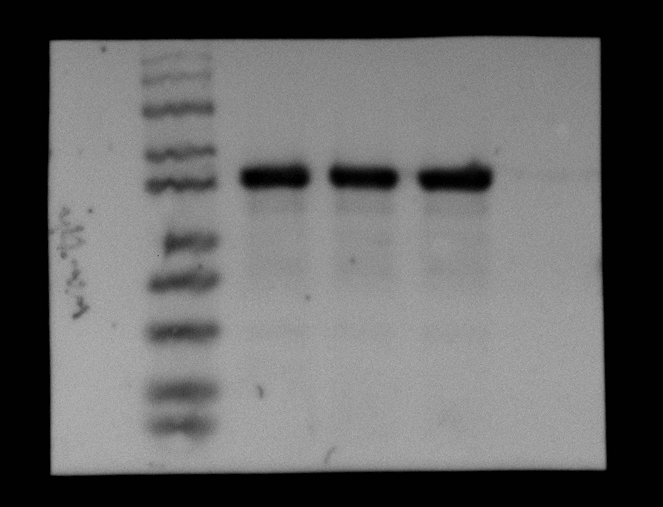
**

**ACTIN**

**
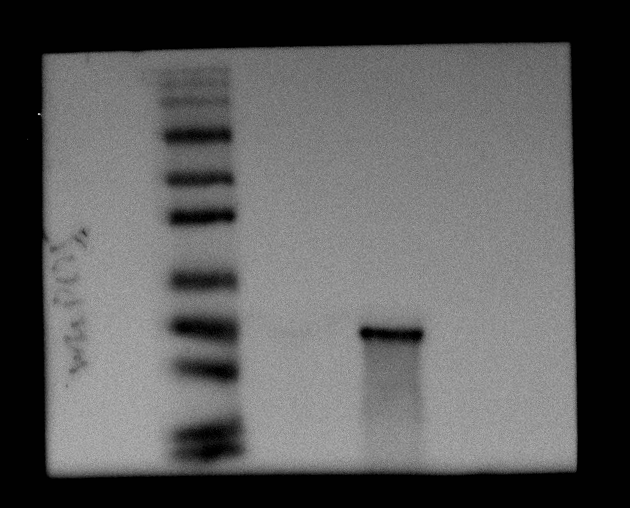
**

**CCDC25**

**
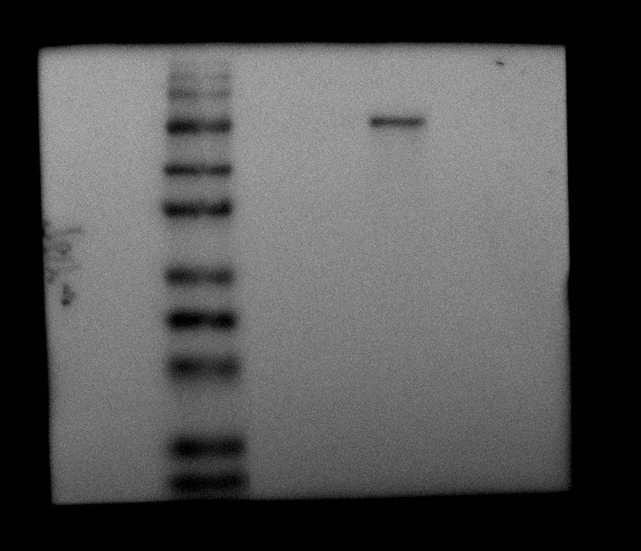
**

**YAP**

**
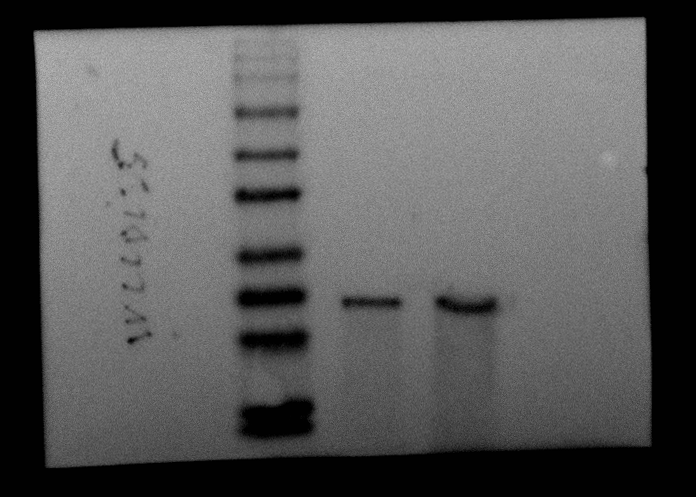
**

**CCDC25**

**
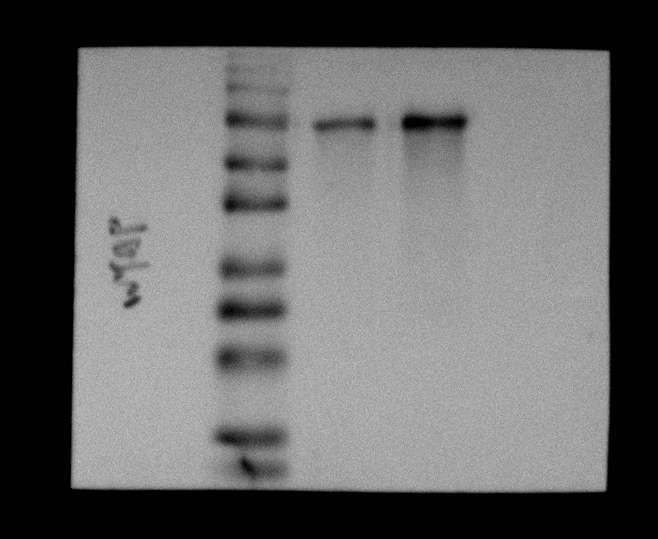
**

**YAP**

**
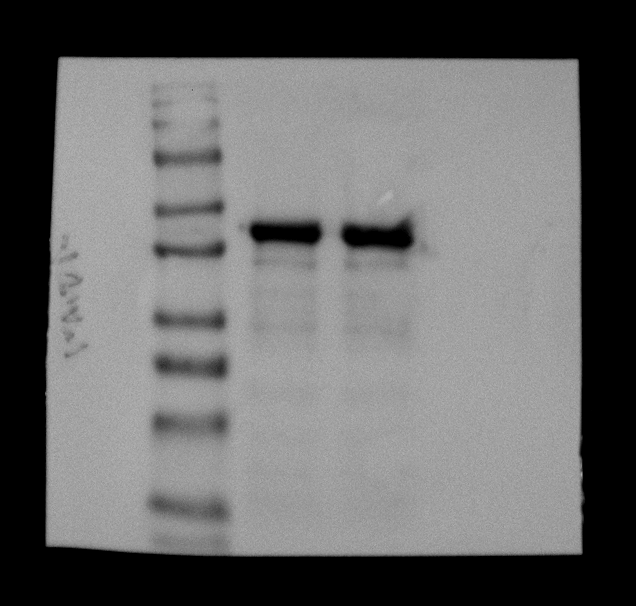
**

**ACTIN**

**
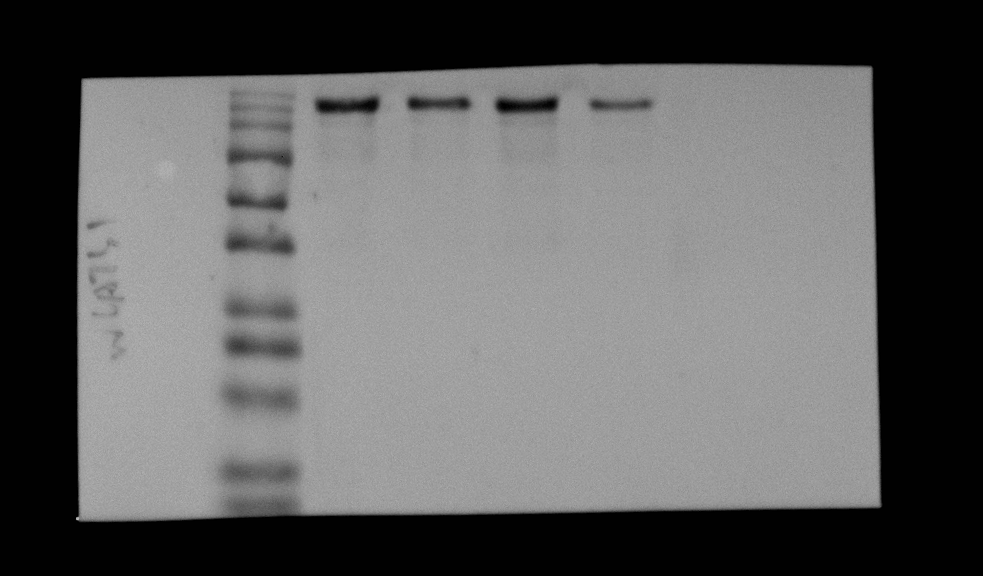
**

**LATS1**

**
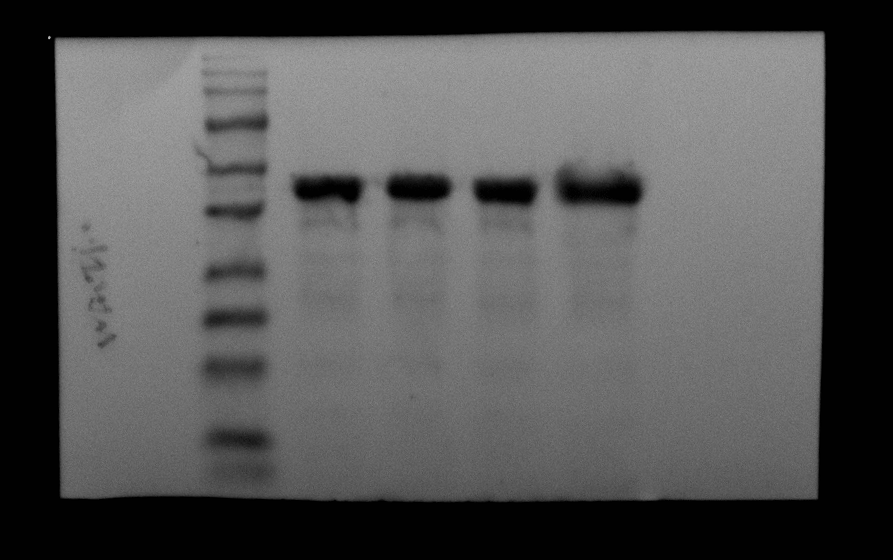
**

**ACTIN**

**
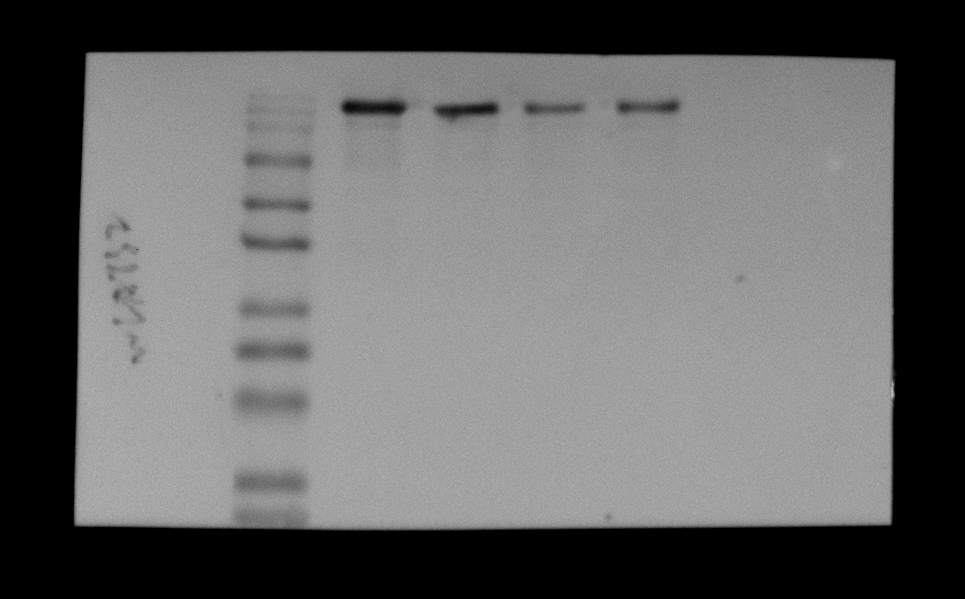
**

**LATS2**

**
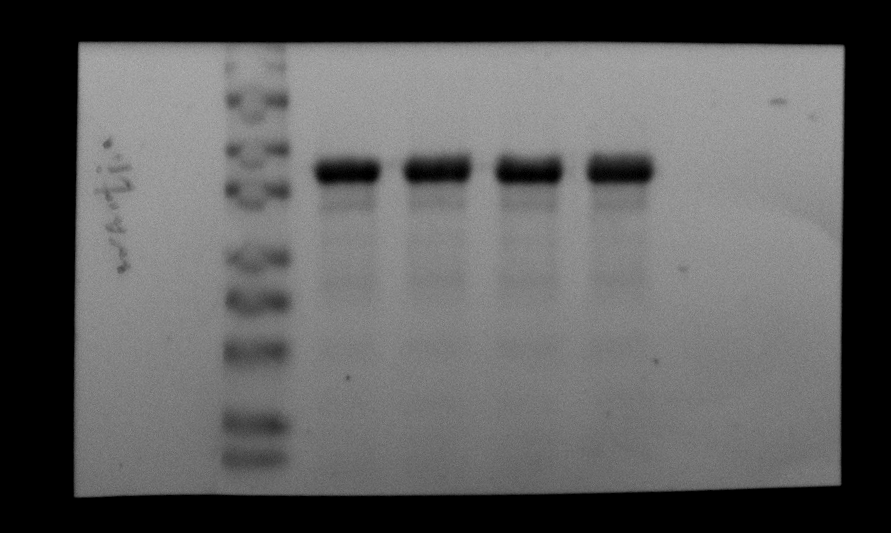
**

**ACTIN**

**
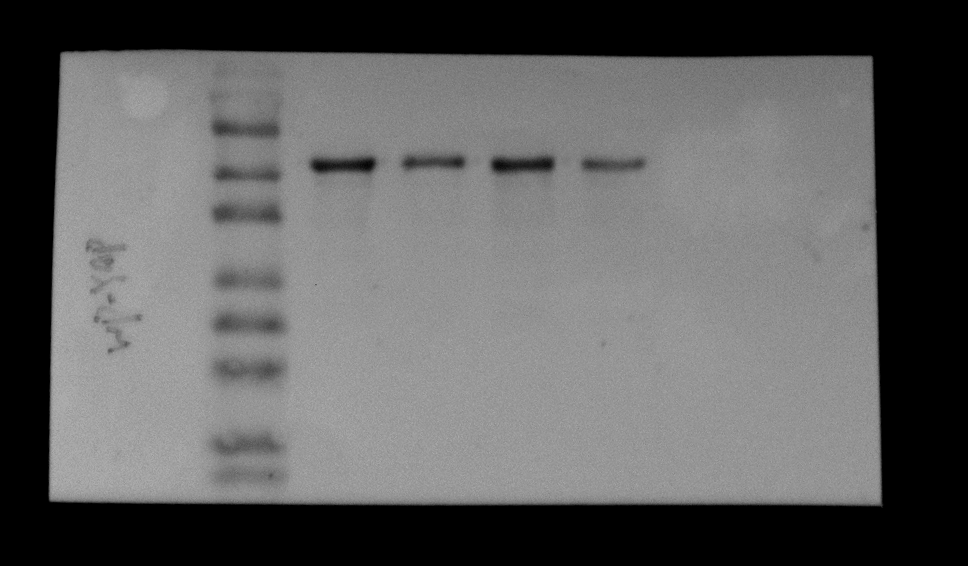
**

**P-YAP**

**
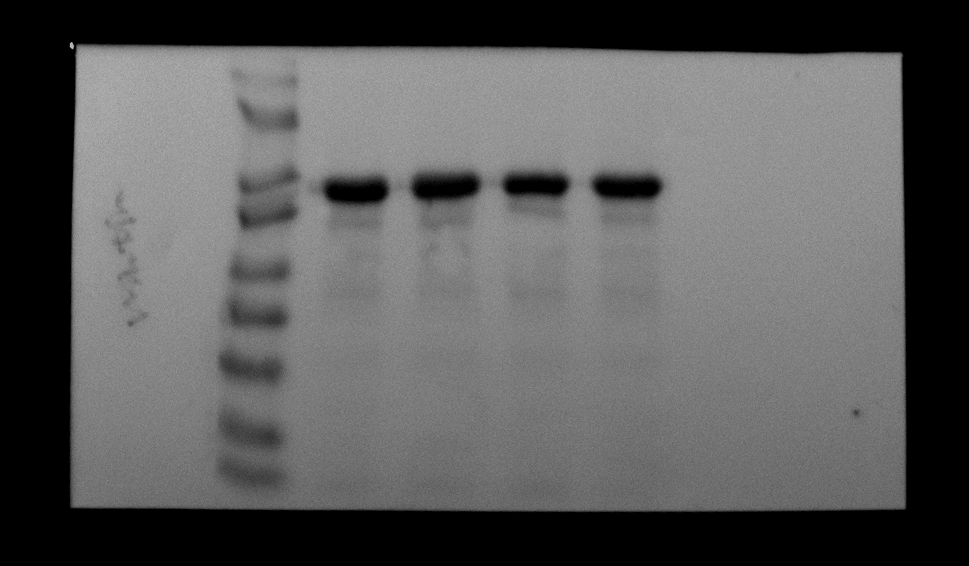
**

**ACTIN**

**
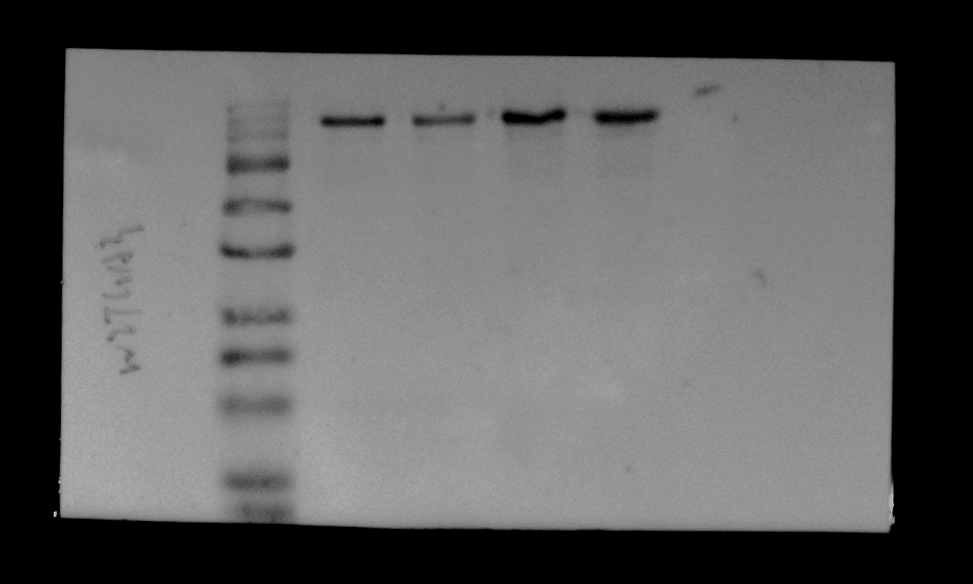
**

**ITGA3**

**
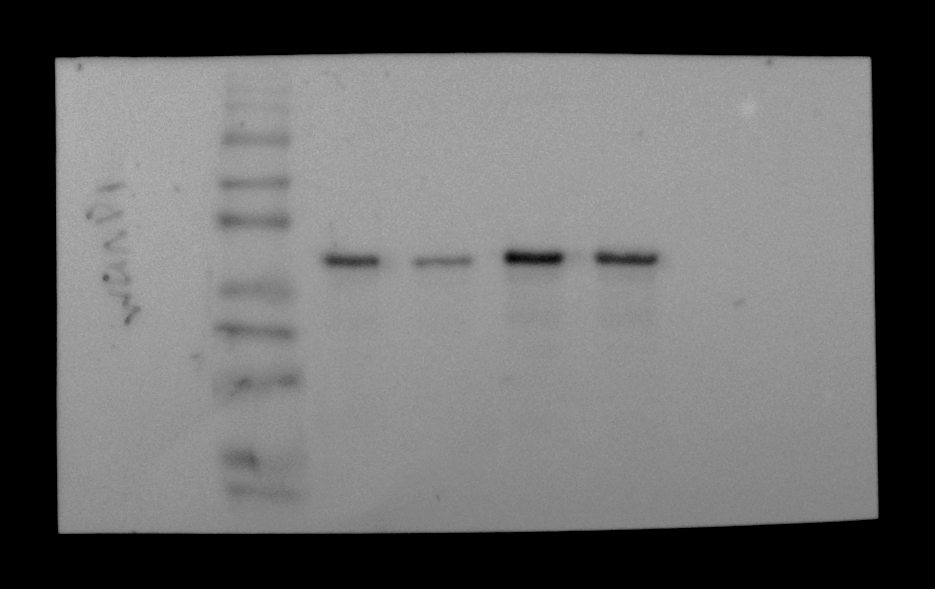
**

**CCND1**

**
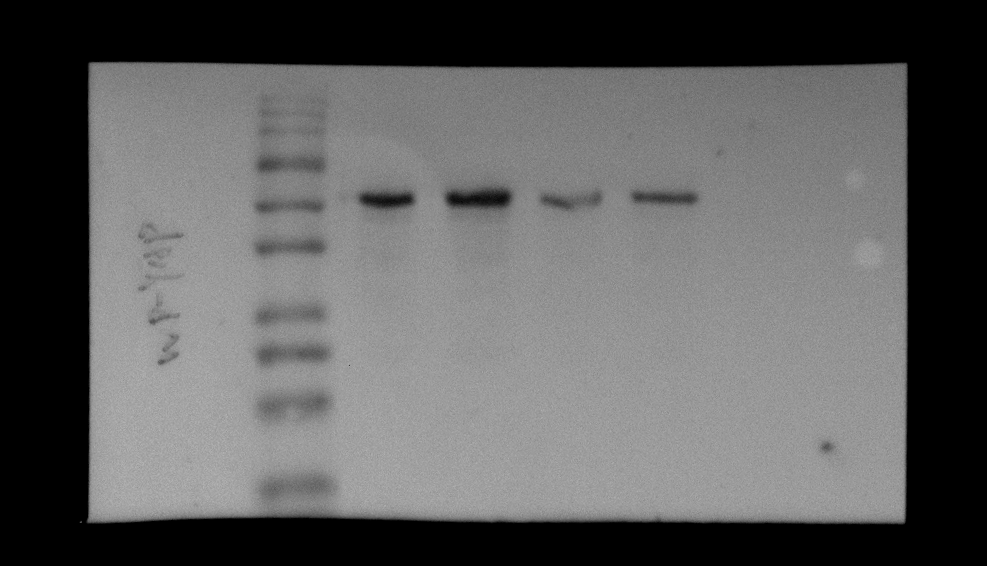
**

**P-YAP**

**
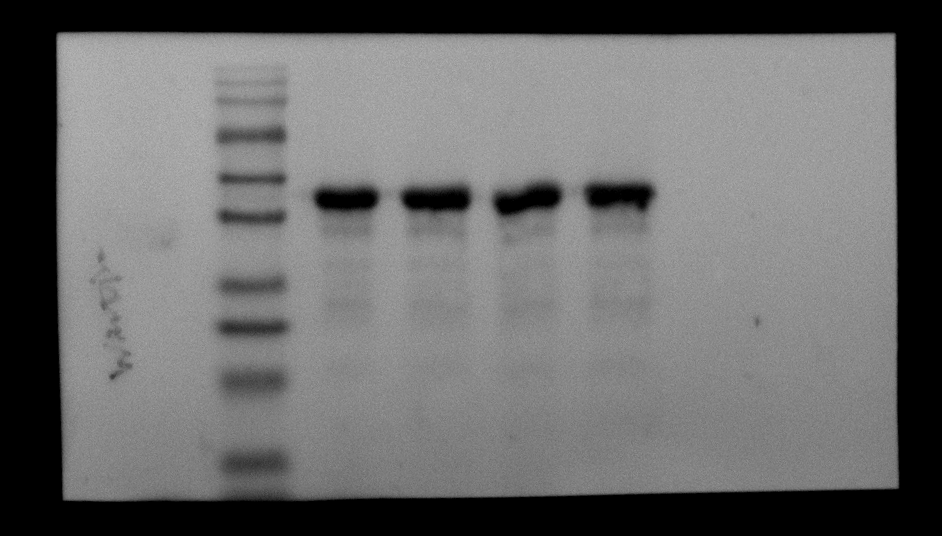
**

**ACTIN**

**
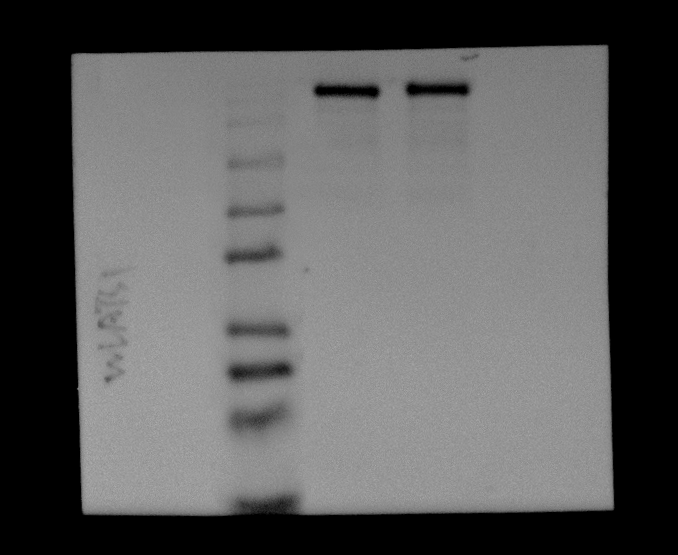
**

**LATS1**

**
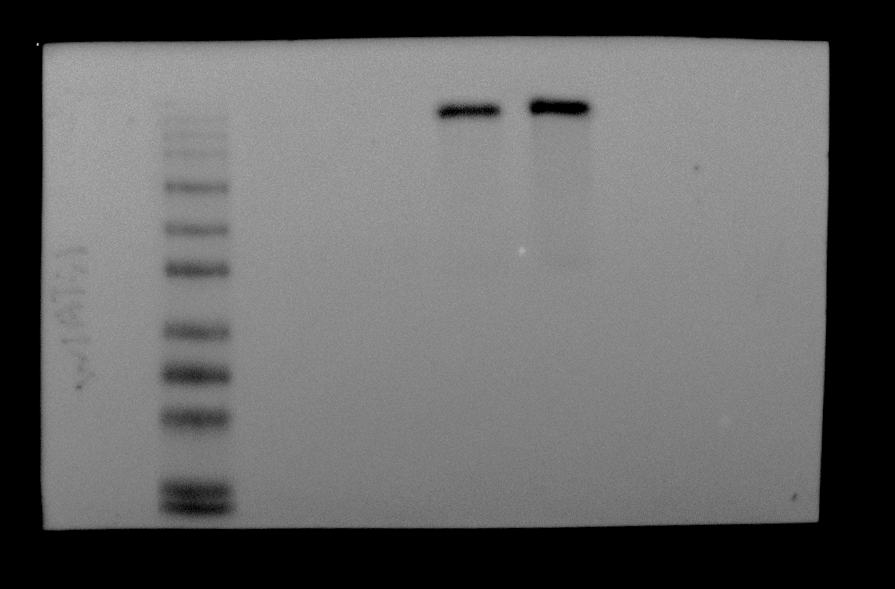
**

**LATS1**

**
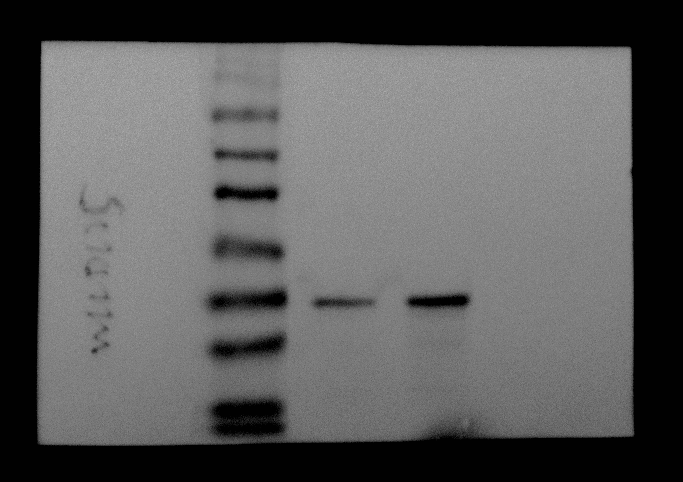
**

**CCDC25**

**
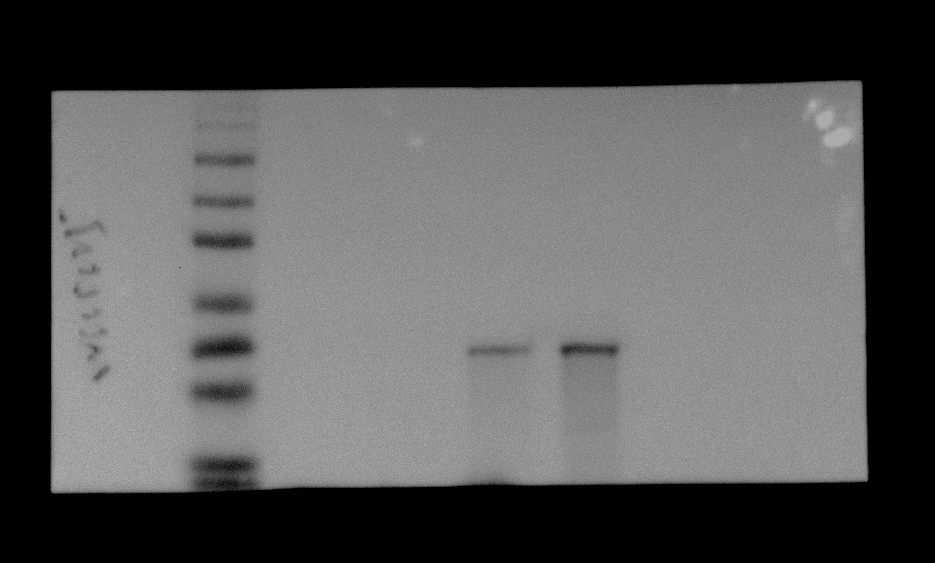
**

**CCDC25**

**
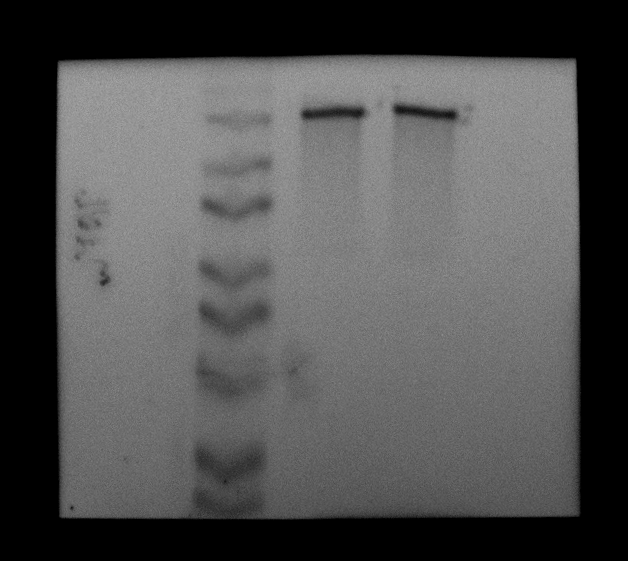
**

**YAP
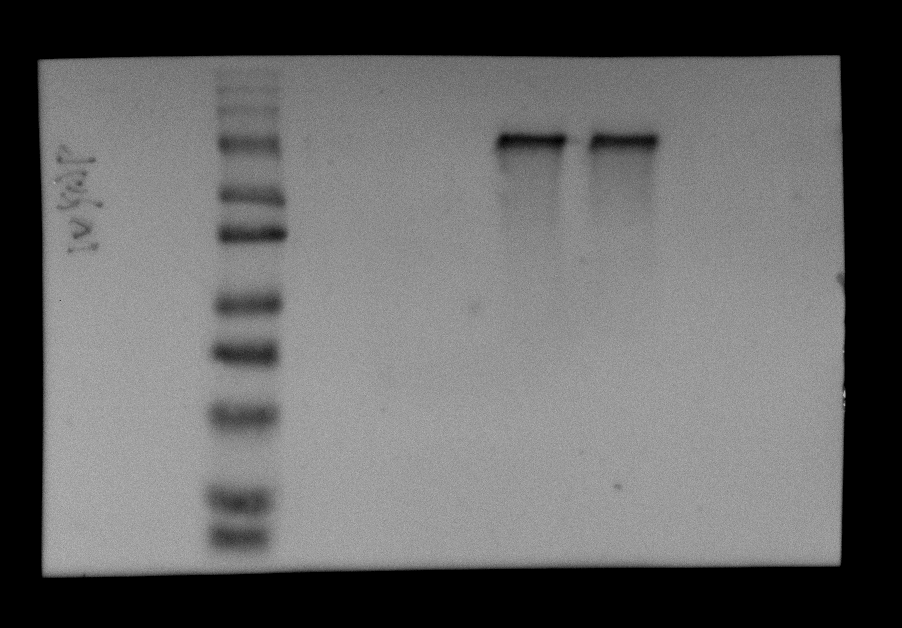
**

**YAP
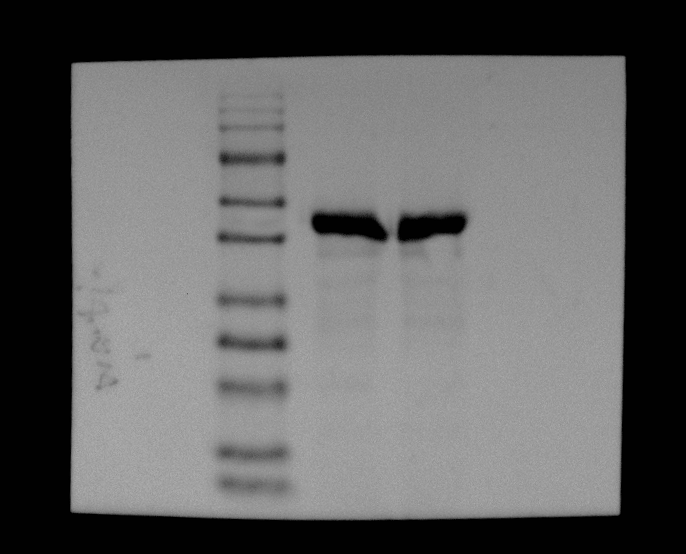
**

**ACTIN**

**
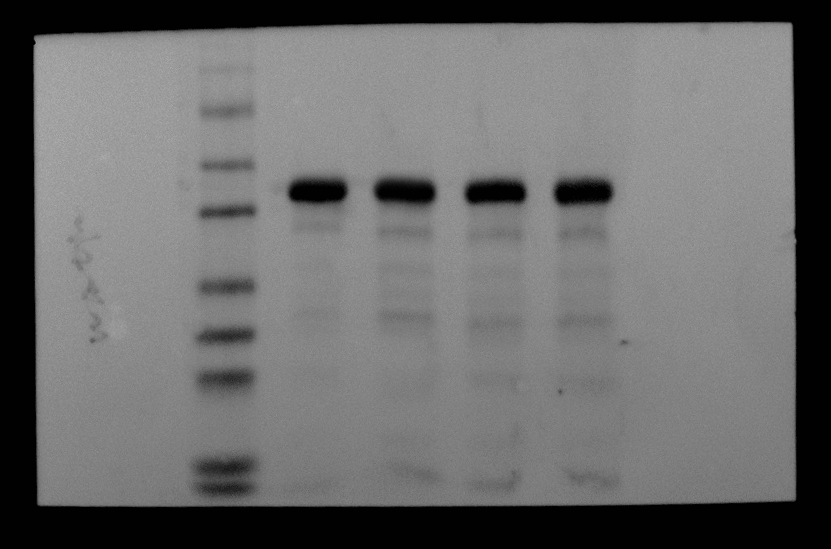
**

**ACTIN**
